# Supplementary material for: Novel, provable algorithms for efficient ensemble-based computational protein design and their application to the redesign of the c-Raf-RBD:KRas protein-protein interface
Source: PLoS Comput Biol. 2020 Jun 8;16(6):e1007447. doi: 10.1371/journal.pcbi.1007447 (PMC7329130; doi:10.1371/journal.pcbi.1007447)
Supplement: S1 Text — (PDF) [file pcbi.1007447.s001.pdf]

Novel, provable algorithms for efficient ensemble-based computational protein design and their application to the redesign of the c-Raf-RBD:KRas protein-protein interface (Supporting information)

Anna U. Lowegard†, Marcel S. Frenkel†, Graham T. Holt, Jonathan D. Jou, Adegoke A. Ojewole, and Bruce R. Donald

† These authors contributed equally to the work.

S1 Text. Homology model of c-Raf-RBD in complex with KRas

PDB ID 4DSN [1] is an X-ray crystal structure of KRas isoform 2B which contains G12D, a mutation that locks KRas into its active form. This structure of KRas bound to a GTP analog was used to model KRas^{GTP}. PDB ID 1GUA [2] is an X-ray crystal structure of c-Raf in complex with Rap, a Ras homolog. These two structures (4DSN and 1GUA) were aligned using PyMol [3]. Rap was then removed, leaving c-Raf poised in complex with KRas from PDB ID 4DSN. This complex was then minimized using Sander from AmberTools for 200 steps [4] to relax any steric clashes.

References

[1] T. Maurer, L. S. Garrenton, A. Oh, K. Pitts, D. J. Anderson, N. J. Skelton, B. P. Fauber, B. Pan, S. Malek, D. Stokoe, et al. Small-molecule ligands bind to a distinct pocket in Ras and inhibit sos-mediated nucleotide exchange activity. *Proceedings of the National Academy of Sciences*, 109(14):5299–5304, 2012.

[2] N. Nassar, G. Horn, C. Herrmann, C. Block, R. Janknecht, and A. Wittinghofer. Ras/Rap effector specificity determined by charge reversal. *Nature Structural and Molecular Biology*, 3(8):723, 1996.

[3] W. L. DeLano. The PyMOL molecular graphics system. *http://www. pymol. org*, 2002.

[4] D. Pearlman, D. Case, J. Caldwell, W. Ross, T. Cheatham, S DeBolt, D Ferguson, G Seibel, and P Kollman. AMBER: a package of computer programs for applying molecular mechanics, normal mode analysis, molecular dynamics and free energy calculations to simulate the structural and energetic properties of molecules. *Comput Phys Commun*, 91(42):1–41, 1995.
