# Supplementary material for: Novel, provable algorithms for efficient ensemble-based computational protein design and their application to the redesign of the c-Raf-RBD:KRas protein-protein interface
Source: PLoS Comput Biol. 2020 Jun 8;16(6):e1007447. doi: 10.1371/journal.pcbi.1007447 (PMC7329130; doi:10.1371/journal.pcbi.1007447)
Supplement: S2 Text — (PDF) [file pcbi.1007447.s002.pdf]

Novel, provable algorithms for efficient ensemble-based computational protein design and their application to the redesign of the c-Raf-RBD:KRas protein-protein interface (Supporting information)

Anna U. Lowegard†, Marcel S. Frenkel†, Graham T. Holt, Jonathan D. Jou, Adegoke A. Ojewole, and Bruce R. Donald

† These authors contributed equally to the work.

S2 Text. Details of the expression and purification of c-Raf-RBD variants

The c-Raf-RBD variants were made in a C81I, C96M background (as described in the Section entitled "Materials and methods"). Each c-Raf-RBD variant was expressed with a N-term His-SUMO tag to increase expression and facilitate purification. Variants were then grown to an OD600 of 0.8 at 37°C in Rosetta 2(DE3) cells in LB media with kanamycin and chloramphenicol. Cells were then induced with 1 mM IPTG at 16°C overnight. Cells were then pelleted, re-suspended in Lysis Buffer (40 mM Tris-HCl, 50 mM NaCl, 5 mM $MgCl_2$, 5 mM $CaCl_2$, and 1 mM TCEP at pH 8.0 with 1 mM PMSF), and lysed with 5 passages through a homo-fluidizer. Next, the lysate was incubated with 5 µl of micrococcal nuclease (stock concentration of 2,000,000 gel units/ml) per liter of cell growth for 20 minutes at 37°C. The lysate was centrifuged and the supernatant was then loaded onto a nickel NTA column in lysis buffer and eluted with a gradient from 0-100% Buffer B (40 mM Tris-HCl, 500 mM NaCl, 5 mM $MgCl_2$, 500 mM Imidazole, and 1 mM TCEP at pH 7.4). Fractions of interest were collected, concentrated (10 kDa MWCO), and buffer exchanged through a G-25 Sephadex column into Protease Buffer (40 mM Tris-HCl and 250 mM NaCl at pH 8.0). Each c-Raf-RBD variant was then concentrated and incubated with 3 mg of SUMO protease (roughly 1 mg per 10 mg of SUMO-labeled c-Raf-RBD) at 30°C overnight to cleave the His-SUMO tag. Each variant was then loaded onto a nickel NTA column in Protease Buffer and eluted with a gradient from 0-100% Buffer B. Flow-through was collected, concentrated, and buffer exchanged through a G-25 Sephadex column into 50 mM phosphate at pH 6.5. Desalted variants were then further purified with a cation exchange SP Sepharose column eluted with 50 mM phosphate, 500 mM NaCl, and 5 mM TCEP at pH 6.5. Each c-Raf-RBD variant was then concentrated (3 kDa MWCO) and buffer exchanged into 40 mM Tris-HCl, 250 mM NaCl, 5 mM $MgCl_2$, and 1 mM TCEP at pH 7.4. The purity of each variant was determined by SDS-PAGE gel (data not shown) and further identified by matrix assisted laser desorption ionization time of flight (MALDI-TOF) mass spectrometry (data not shown).
