# Supplementary material for: Novel, provable algorithms for efficient ensemble-based computational protein design and their application to the redesign of the c-Raf-RBD:KRas protein-protein interface
Source: PLoS Comput Biol. 2020 Jun 8;16(6):e1007447. doi: 10.1371/journal.pcbi.1007447 (PMC7329130; doi:10.1371/journal.pcbi.1007447)
Supplement: S3 Text — (PDF) [file pcbi.1007447.s003.pdf]

Novel, provable algorithms for efficient ensemble-based computational protein design and their application to the redesign of the c-Raf-RBD:KRas protein-protein interface (Supporting information)

Anna U. Lowegard†, Marcel S. Frenkel†, Graham T. Holt, Jonathan D. Jou, Adegoke A. Ojewole, and Bruce R. Donald

† These authors contributed equally to the work.

S3 Text. Details of the expression and purification of KRas

KRas was made with a N-term His-tag in a C118S background, which was introduced to increase expression and stability (see the Section entitled "Materials and methods"). KRas was grown to an OD600 of 0.8 at 37°C in Rosetta 2(DE3) cells in LB media with kanamycin and chloramphenicol. Cells were then induced with 1 mM IPTG and incubated 37°C for 5 hours before being lowered to 16°C overnight. Cells were then pelleted, re-suspended in Lysis Buffer (40 mM Tris-HCl, 50 mM NaCl, 5 mM $MgCl_2$, 5 mM $CaCl_2$, and 1 mM TCEP at pH 8.0 with 1 mM PMSF) and then lysed with 5 passages through a homo-fluidizer. Next, the lysate was incubated with 5 µl of micrococcal nuclease (stock concentration of 2,000,000 gel units/ml) per liter of cell growth for 20 minutes at 37°C. The lysate was centrifuged and the supernatant was then loaded onto a nickel NTA column in lysis buffer and eluted with a gradient from 0-100% Buffer B (40 mM Tris-HCl, 500 mM NaCl, 5 mM $MgCl_2$, 500 mM Imidazole, and 1 mM TCEP at pH 7.4). Fractions of interest were collected, concentrated (10 kDa MWCO), and buffer exchanged through a G-25 Sephadex column into Desalting Buffer (50 mM Tris-HCl and 50 mM NaCl at pH 8.0). Then, KRas was concentrated (10 kDa MWCO) in preparation for nucleotide loading. The Desalting Buffer was then supplemented with 200 mM ammonium sulphate, 1 µM zinc chloride, 50 units of calf intestinal phosphatase (CIP), and 10 molar excess of a non-hydrolysable GTP analogue guanosine-5'-[(β,γ)-imido]triphosphate (GppNHp). KRas was incubated with GppNHp for 2 hours at room temperature and then overnight at 4°C. The reaction was terminated by the addition of 60 mM $MgCl_2$. Next, $KRas^{GppNHp}$ was loaded onto a Superdex 75 column in 40 mM Tris-HCl, 250 mM NaCl, 5 mM $MgCl_2$, and 1 mM TCEP at pH 7.4. Purity was confirmed by SDS-PAGE gel (data not shown) and GppNHp loading was confirmed by high-performance anion exchange chromatography (HPAEC, data not shown).
