# Supplementary material for: Novel, provable algorithms for efficient ensemble-based computational protein design and their application to the redesign of the c-Raf-RBD:KRas protein-protein interface
Source: PLoS Comput Biol. 2020 Jun 8;16(6):e1007447. doi: 10.1371/journal.pcbi.1007447 (PMC7329130; doi:10.1371/journal.pcbi.1007447)
Supplement: S4 Text — (PDF) [file pcbi.1007447.s004.pdf]

Novel, provable algorithms for efficient ensemble-based computational protein design and their application to the redesign of the c-Raf-RBD:KRas protein-protein interface (Supporting information)

Anna U. Lowegard†, Marcel S. Frenkel†, Graham T. Holt, Jonathan D. Jou, Adegoke A. Ojewole, and Bruce R. Donald

† These authors contributed equally to the work.

S4 Text. Sequence and partition function approximation algorithms.

*K** is a partition function approximation algorithm that approximates a single partition function q_{pl}, q_p, or q_l for a single sequence [1–3]. Historically we have overloaded the term *K** to refer to both the partition function approximation algorithm and the ratio of partition functions: q_{pl}/q_pq_l. The *BBK** algorithm implements a search strategy over sequence space to find the best sequences ranked by this score. In doing so, *BBK** calls the *K** partition function approximation algorithm as a subroutine. In contrast, in the new work herein, *EWAK** is designed as a replacement for the *K** partition function approximation algorithm. Therefore, we compared *BBK** with *K** to *BBK** with *EWAK** for testing purposes.

FRIES is effectively a preprocessing algorithm that is agnostic to the method of partition function calculation, and therefore is run before *BBK**.

References

[1] R. H. Lilien, B. W. Stevens, A. C. Anderson, and B. R. Donald. A novel ensemble-based scoring and search algorithm for protein redesign and its application to modify the substrate specificity of the gramicidin synthetase a phenylalanine adenylation enzyme. *J Comput Biol*, 12(6):740–61, 2005. DOI: 10.1089/cmb.2005.12.740.

[2] I. Georgiev, R. H. Lilien, and B. R. Donald. The minimized dead-end elimination criterion and its application to protein redesign in a hybrid scoring and search algorithm for computing partition functions over molecular ensembles. *J Comput Chem*, 29(10):1527–42, 2008. DOI: 10.1002/jcc.20909.

[3] K. E. Roberts, P. R. Cushing, P. Boisguerin, D. R. Madden, and B. R. Donald. Computational design of a PDZ domain peptide inhibitor that rescues CFTR activity. *PLoS Comput Biol*, 8(4):e1002477, 2012. DOI: 10.1371/journal.pcbi.1002477.
