# Supplementary material for: Novel, provable algorithms for efficient ensemble-based computational protein design and their application to the redesign of the c-Raf-RBD:KRas protein-protein interface
Source: PLoS Comput Biol. 2020 Jun 8;16(6):e1007447. doi: 10.1371/journal.pcbi.1007447 (PMC7329130; doi:10.1371/journal.pcbi.1007447)
Supplement: S5 Text — (PDF) [file pcbi.1007447.s005.pdf]

Novel, provable algorithms for efficient ensemble-based computational protein design and their application to the redesign of the c-Raf-RBD:KRas protein-protein interface (Supporting information)
Anna U. Lowegard[†], Marcel S. Frenkel[†], Graham T. Holt, Jonathan D. Jou, Adegoke A. Ojewole, and Bruce R. Donald

[†] These authors contributed equally to the work.

S5 Text. The relationship between the energy window stopping criterion and epsilon.

The energy window E_w and epsilon values referred to in this manuscript are related, since increasing the energy threshold *must* decrease the value of epsilon, and vice versa. Unfortunately, the precise function relating these two values is not simple to calculate. This function not only depends on the protein state for which we are approximate the partition function, but also requires computing the partition function to at least ε_w or E_w. To see this, consider the following:

Let E_w be the user-specified energy window, and let ε_w be the corresponding value of epsilon. Let c^* represent the global minimum energy conformation (GMEC), and let E be the energy function. Let $Z^\oplus$ and $Z^\ominus$ be the upper and lower bounds on the partition function (definitions based on [1]), respectively, that correspond to the energy window and epsilon values. Let C and U be the sets of conformations with energies below and above $E(c^*) + E_w$, respectively. Note that c^* is necessarily a member of C. As usual, let R and T be the gas constant and temperature. We can derive a relationship between E_w and ε_w, as follows.

$$\begin{aligned}
\varepsilon_w &= \frac{Z^\oplus - Z^\ominus}{Z^\ominus} \\
&= \frac{Z^\oplus}{Z^\ominus} - 1 \\
&= \frac{\sum\limits_{c \in C} \exp(-E(c)/RT) + |U| \exp\left(-\left(E(c^*) + E_w\right)/RT\right)}{\sum\limits_{c \in C} \exp(-E(c)/RT)} - 1 \\
&= \frac{|U| \exp\left(-\left(E(c^*) + E_w\right)/RT\right)}{\sum\limits_{c \in C} \exp(-E(c)/RT)}
\end{aligned}$$

To solve or approximate this relationship, we would need to know at least the number of conformations above and below the energy threshold $E(c^*) + E_w$. Counting the number of discrete protein conformations below some specified energy threshold has been shown to be #P-complete [2]. Therefore, deriving the ε_w associated with a particular E_w requires actually computing the partition function to E_w.

References

[1] R. H. Lilien, B. W. Stevens, A. C. Anderson, and B. R. Donald. A novel ensemble-based scoring and search algorithm for protein redesign and its application to modify the substrate specificity of the gramicidin synthetase a phenylalanine adenylation enzyme. *J Comput Biol*, 12(6):740–61, 2005. DOI: `10.1089/cmb.2005.12.740`.

[2] H. Nisonoff. Efficient partition function estimation in computational protein design: probabalistic guarantees and characterization of a novel algorithm. *B.S. Thesis. Department of Mathematics, Duke University. http://hdl.handle.net/10161/9746*, 2015.
