# Supplementary material for: Novel, provable algorithms for efficient ensemble-based computational protein design and their application to the redesign of the c-Raf-RBD:KRas protein-protein interface
Source: PLoS Comput Biol. 2020 Jun 8;16(6):e1007447. doi: 10.1371/journal.pcbi.1007447 (PMC7329130; doi:10.1371/journal.pcbi.1007447)
Supplement: S1 Table — Each protein structure has its PDB ID listed along with its molecule names as presented in the Protein Database entry for each structure. Individual designs are not listed or described here, but the necessary code and data is provided for the interested reader (see Data availability statement). (PDF) [file pcbi.1007447.s006.pdf]

Novel, provable algorithms for efficient ensemble-based computational protein design and their application to the redesign of the c-Raf-RBD:KRas protein-protein interface (Supporting information)

Anna U. Lowegard†, Marcel S. Frenkel†, Graham T. Holt, Jonathan D. Jou, Adegoke A. Ojewole, and Bruce R. Donald

† These authors contributed equally to the work.

S1 Table. Protein structures used in computational experiments as described in the Section entitled "Computational experiments."

Each protein structure has its PDB ID listed along with its molecule names as presented in the Protein Database entry for each structure. Individual designs are not listed or described here, but the necessary code and data is provided for the interested reader (see "Data availability statement").

PDB ID	Molecule(s)
2RL0	Fibronectin and Fibronectin-binding protein
4WEM	K88 fimbrial protein AC and Anti-F4+ETEC bacteria VHH variable region
2P4A	Ribonuclease pancreatic and ANTIBODY CAB-RN05
2RFE	Epidermal growth factor receptor and ERBB receptor feedback inhibitor 1
2RF9	Epidermal growth factor receptor and ERBB receptor feedback inhibitor 1
4WWI	Immunoglobulin G-binding protein A and Ig gamma-3 chain C region
4ZNC	Immunoglobulin G-binding protein A and Ig gamma-3 chain C region
3U7Y	NIH45-46 heavy chain, Ig gamma-1 chain C region, Envelope glycoprotein gp160, and NIH45-46 light chain, Ig kappa chain C region
2HNV	Oxytocin-neurophysin 1
4Z80	EGF family domain-containing protein and Cytoadherence-linked asexual protein
4U3S	Cellulosomal scaffoldin adaptor protein B and Cellulosomal scaffoldin
1B6C	FK506-BINDING PROTEIN and TGF-B SUPERFAMILY RECEPTOR TYPE I
3GXC	Ephrin type-A receptor 4 and Ephrin-B2
1GWC	GLUTATHIONE S-TRANSFERASE TSI-1
4WYU	Protein scribble homolog and peptide SER-TRP-PHE-GLN-THR-ASP-LEU
5IT3	Lysine-specific histone demethylase 1A
2HNU	Oxytocin-neurophysin 1
5D68	Krev interaction trapped protein 1
5A6Y	FUCOSE-BINDING LECTIN PA-IIL
3K3Q	Llama Aa1 VHH domain and Botulinum neurotoxin type A
3CAL	Fibronectin and peptide from Fibronectin-binding protein A
1A0R	TRANSDUCIN (BETA SUBUNIT), TRANSDUCIN (GAMMA SUBUNIT), and PHOSDUCIN
5EM2	Ribosome biogenesis protein ERB1 and Ribosome biogenesis protein YTM1
4PXF	Rhodopsin and S-arrestin
3EB6	Baculoviral IAP repeat-containing protein 3 and Ubiquitin-conjugating enzyme E2 D2
3BU8	Telomeric repeat-binding factor 2 and TERF1-interacting nuclear factor 2
5DC0	Fibronectin and Tyrosine-protein kinase ABL1
2Q1E	Amyloidogenic immunoglobulin light chain protein AL-09
2XXM	CAPSID PROTEIN P24, CAMELID VHH 9, and INHIBITOR OF CAPSID ASSEMBLY
2Q2A	ArtJ
2RFD	Epidermal growth factor receptor and ERBB receptor feedback inhibitor 1
5DC4	Tyrosine-protein kinase ABL1 and AS25 monobody
2XGY	RELIK CAPSID N-TERMINAL DOMAIN and PEPTIDYL-PROLYL CIS-TRANS ISOMERASE A
2P49	Ribonuclease pancreatic and ANTIBODY CAB-RN05
3RJQ	C186 gp120 and Llama VHH A12
