# Supplementary material for: Novel, provable algorithms for efficient ensemble-based computational protein design and their application to the redesign of the c-Raf-RBD:KRas protein-protein interface
Source: PLoS Comput Biol. 2020 Jun 8;16(6):e1007447. doi: 10.1371/journal.pcbi.1007447 (PMC7329130; doi:10.1371/journal.pcbi.1007447)
Supplement: S2 Table — For each listed c-Raf-RBD variant, we give the experimental percent change in KRas binding relative to wild-type c-Raf-RBD as reported in [48] (no Ka values were reported in [48] so the corresponding entries are left blank here) and as calculated from reported binding values in [54] and [60] (reported here as Exp. Kd), the EWAK* computationally predicted percent change in binding, the Δb values as described in the Section entitled “fries/EWAK* retrospectively predicted the affect mutations in c-Raf-RBD have on binding to KRas,” and the rankings that correspond to these values. The Δb values are calculated as follows: log10(%) − 2 where % represents the percent change in binding upon mutation. The rankings have a Pearson correlation of 0.81. The Pearson correlation between the change in binding values Δb is 0.64. (PDF) [file pcbi.1007447.s007.pdf]

Novel, provable algorithms for efficient ensemble-based computational protein design and their application to the redesign of the c-Raf-RBD:KRas protein-protein interface (Supporting information)

Anna U. Lowegard†, Marcel S. Frenkel†, Graham T. Holt, Jonathan D. Jou, Adegoke A. Ojewole, and Bruce R. Donald

† These authors contributed equally to the work.

S2 Table. Experimental and computational percent change in binding and rankings.

For each listed c-Raf-RBD variant, we give the experimental percent change in KRas binding relative to wild-type c-Raf-RBD as reported in [1] (no K_a values were reported in [1] so the corresponding entries are left blank here) and as calculated from reported binding values in [2] and [3] (reported here as Exp. K_d), the *EWAK** computationally predicted percent change in binding, the Δb values as described in the Section entitled "FRIES/EWAK* retrospectively predicted the affect mutations in c-Raf-RBD have on binding to KRas," and the rankings that correspond to these values. The Δb values are calculated as follows: $\log_{10}(\%) - 2$ where $\%$ represents the percent change in binding upon mutation. The rankings have a Pearson correlation of 0.81. The Pearson correlation between the change in binding values Δb is 0.64.

Mutation(s)	Exp. $K_d(\mu M)$	Exp. (%)	Δb (Exp.)	Comp. (%)	Δb (Comp.)	Exp. Ranking	Comp. Ranking
Wild-Type	0.13	100.00	0.00	100.00	0.00	N/A	N/A
R89L		1.3×10^{-7}	-8.89	1.64×10^{-10}	-11.79	1	3
F61W/R67L/V69E/N71R/V88I/A85K	57.0	0.23	-2.64	2.20×10^{-11}	-12.66	2	2
K84A	14.0	0.93	-2.03	3.03×10^{-5}	-6.52	3	4
Q66A	7.40	1.76	-1.75	0.99	-2.00	4	16
A85D		3.00	-1.52	0.01	-4.00	5	10
R59A	3.80	3.42	-1.47	8.09×10^{-4}	-5.09	6	7
F61W/V69E/N71R/V88I	2.80	4.64	-1.33	0.03	-3.56	7	12
R67A	2.10	6.19	-1.21	1.78×10^{-4}	-5.75	8	6
K84L		8.60	-1.07	1.56×10^{-4}	-5.81	9	5
Q66K		9.00	-1.05	1.78	-1.75	10	18
T68A	1.30	10.00	-1.00	4.03	-1.39	11	20
V88D		10.00	-1.00	0.05	-3.34	12	13
T68K		11.00	-0.96	5.63×10^{-17}	-18.3	13	1
V69A	0.95	13.68	-0.86	5.08	-1.29	14	22
A85I		18.00	-0.74	8.64	-1.06	15	24
K65A	0.70	18.57	-0.73	1.04	-1.98	16	17
K65E	0.67	19.40	-0.71	0.71	-2.15	17	15
N64A	0.61	21.31	-0.67	5.77	-1.24	18	23
V69R		29.00	-0.54	1.40×10^{4}	2.15	19	34
K87Q		30.00	-0.52	12.43	-0.91	20	26
K65M	0.41	31.71	-0.50	2.53	-1.60	21	19
N71E		34.00	-0.47	0.15	-2.84	22	14
F61W	0.36	36.11	-0.44	116.20	0.07	23	29
F61W/R67L/N71R/V88I	0.36	36.11	-0.44	0.01	-3.97	24	11
V88I	0.33	39.39	-0.40	16.15	-0.79	25	28
R67L		42.00	-0.38	3.06×10^{-3}	-4.51	26	9
R59L		43.00	-0.37	2.51×10^{-3}	-4.60	27	8
K84R		49.00	-0.31	10.01	-1.00	28	25
N64D		50.00	-0.30	15.60	-0.81	29	27
F61W/N71R/V88I	0.24	54.17	-0.27	1.96×10^{4}	2.29	30	35
K87R		100.00	0.00	120.04	0.08	31	30
F61W/N71R	0.08	162.50	0.21	1.10×10^{5}	3.04	32	37
V88K		171.00	0.23	127.37	0.11	33	31
V88H		266.00	0.42	227.92	0.36	34	32
A85R		290.00	0.46	1.50×10^{7}	5.17	35	39
N71R	0.04	325.00	0.51	9.97×10^{4}	3.00	36	36
N64K		380.00	0.58	4.47	-1.35	37	21
V88R		400.00	0.60	2.44×10^{3}	1.39	38	33
A85K/V88R		550.00	0.74	1.33×10^{7}	5.12	39	38
A85K		700.00	0.85	2.13×10^{7}	5.33	40	40
N71R/A85K	0.015	866.67	0.94	3.63×10^{9}	7.56	41	41

References

[1] M. Fridman, H. Maruta, J. Gonez, F. Walker, H. Treutlein, J. Zeng, and A. Burgess. Point mutants of c-raf-1 RBD with elevated binding to v-Ha-Ras. *Journal of Biological Chemistry*, 275(39):30363–30371, 2000.

[2] C. Block, R. Janknecht, C. Herrmann, N. Nassar, and A. Wittinghofer. Quantitative structure-activity analysis correlating Ras/Raf interaction in vitro to Raf activation in vivo. *Nature structural biology*, 3(3):244, 1996.

[3] D. Filchtinski, O. Sharabi, A. Rüppel, I. R. Vetter, C. Herrmann, and J. M. Shifman. What makes Ras an efficient molecular switch: a computational, biophysical, and structural study of Ras-GDP interactions with mutants of Raf. *Journal of molecular biology*, 399(3):422–435, 2010.
