# Supplementary material for: Novel, provable algorithms for efficient ensemble-based computational protein design and their application to the redesign of the c-Raf-RBD:KRas protein-protein interface
Source: PLoS Comput Biol. 2020 Jun 8;16(6):e1007447. doi: 10.1371/journal.pcbi.1007447 (PMC7329130; doi:10.1371/journal.pcbi.1007447)
Supplement: S3 Table — Each section of the table shows the results of the redesign of a residue position in c-Raf-RBD in the c-Raf-RBD:KRas PPI in order of increasing upper bound on log(K*). The table contains the values for upper and lower bounds on log(K*) values (these bounds are described in detail in [32]). *Design results for the wild-type amino acid identity for each position. †Mutations that were selected for experimental testing and validation. (PDF) [file pcbi.1007447.s008.pdf]

Novel, provable algorithms for efficient ensemble-based computational protein design and their application to the redesign of the c-Raf-RBD:KRas protein-protein interface (Supporting information)

Anna U. Lowegard†, Marcel S. Frenkel†, Graham T. Holt, Jonathan D. Jou, Adegoke A. Ojewole, and Bruce R. Donald

† These authors contributed equally to the work.

S3 Table. Table of computational predictions for point mutants in c-Raf-RBD.

Each section of the table shows the results of the redesign of a residue position in c-Raf-RBD in the c-Raf-RBD:KRas PPI in order of increasing upper bound on $\log(K^*)$. The table contains the values for upper and lower bounds on $\log(K^*)$ values (these bounds are described in detail in [1]).
*Design results for the wild-type amino acid identity for each position.
†Mutations that were selected for experimental testing and validation.

**Mutation**	**Lower Bound** log (K^*)	**Upper Bound** log (K^*)
T57E	1.47	1.50
T57D	2.05	2.07
T57G	3.34	3.35
T57Q	3.38	3.42
T57M†	3.43	3.46
T57A	3.47	3.48
T57S	3.45	3.53
T57F	3.61	3.63
T57C	3.63	3.66
T57Y	3.60	3.66
T57L	3.68	3.71
T57N	3.71	3.77
T57Hid	3.72	3.81
T57V	3.78	3.81
T57I	3.86	3.90
T57*	3.82	3.92
T57W	3.97	4.02
T57Hie	4.22	4.26
T57K†	5.01	5.07
T57R	5.03	5.12
T57Hip	5.62	5.70
R59D	9.48	9.74
R59E	10.30	10.64
R59G	12.94	13.03
R59A	12.96	13.06
R59N	12.88	13.10

R59V	13.01	13.11
R59C	12.96	13.14
R59S	12.88	13.15
R59F	12.96	13.15
R59Y	12.99	13.18
R59T	12.96	13.25
R59I	13.06	13.29
R59Hid	13.03	13.35
R59L	13.42	13.58
R59M	13.28	13.68
R59Q	13.55	13.74
R59W	13.93	14.04
R59Hie	13.88	14.25
R59Hip	15.86	16.21
R59K	16.02	16.58
R59*	17.85	18.30
K65T	6.94	7.91
K65G	8.46	8.89
K65D	8.47	9.08
K65A	8.73	9.10
K65E	8.56	9.11
K65L	8.91	9.38
K65S	8.79	9.38
K65I	9.13	9.52
K65N	9.06	9.55
K65C	9.05	9.57
K65Q	9.04	9.57
K65F	9.06	9.68
K65M	9.09	9.70
K65Y	9.10	9.77
K65Hip	9.40	9.88
K65W	9.21	9.90
K65Hid	9.36	9.92
K65Hie	9.42	10.02
K65R	10.46	11.31
K65*	10.62	11.48
Q66Hie	2.76	2.82
Q66Hip	3.17	3.23
Q66L	7.17	7.25
Q66E	7.93	7.95
Q66Hid	8.64	8.71
Q66D	11.37	11.42

Q66A	11.44	11.48
Q66G	11.02	11.57
Q66S	11.59	11.71
Q66K	11.63	11.81
Q66N	11.79	11.85
Q66R	11.87	11.96
Q66C	12.43	12.47
Q66T	12.39	12.48
Q66M	12.65	12.73
Q66*	13.43	13.49
R67Y	7.88	8.43
R67E	8.58	9.11
R67D	8.44	9.21
R67W	9.28	9.62
R67F	10.74	11.14
R67Hie	11.62	12.31
R67G	12.14	12.39
R67A	12.37	12.61
R67S	12.33	12.98
R67Hid	12.59	13.19
R67C	12.85	13.20
R67T	12.73	13.21
R67Q	12.82	13.32
R67N	12.64	13.33
R67V	13.12	13.46
R67M	12.83	13.51
R67L	13.63	13.91
R67I	13.59	14.10
R67Hip	14.99	15.62
R67K	16.92	17.61
R67*	17.94	18.59
T68Q	−12.00	−11.60
T68R	−8.92	−8.34
T68E	−2.55	−2.23
T68K	−2.20	−1.80
T68M	−0.92	−0.62
T68I	2.82	2.91
T68Hid	4.01	4.21
T68Hie	6.16	6.38
T68D	6.54	6.74
T68Hip	7.04	7.23
T68V	10.03	10.10

T68N	11.61	11.94
T68G	14.43	14.46
T68A	14.75	14.79
T68C	14.84	14.95
T68S	14.91	15.17
T68*	16.04	16.21
V69Y	−20.72	−18.18
V69W	−3.45	−1.01
V69F	−0.74	1.54
V69Hie	17.19	19.15
V69E	18.66	19.83
V69Hid	18.00	19.84
V69L	19.83	21.10
V69D	19.91	21.16
V69G	21.61	22.33
V69A	22.34	23.03
V69I	22.03	23.10
V69Hip	21.19	23.32
V69S	22.33	23.37
V69N	22.27	23.43
V69C	22.79	23.61
V69T	22.76	23.70
V69Q	22.85	23.89
V69*	23.67	24.30
V69M	23.30	24.48
V69K	24.95	26.48
V69R	25.56	27.16
N71E	4.31	4.71
N71D	5.70	5.95
N71G	6.86	6.96
N71A	7.00	7.11
N71S	7.02	7.22
N71I	6.91	7.26
N71C	7.14	7.30
N71Hid	7.05	7.32
N71T	7.18	7.46
N71*	7.25	7.49
N71V	7.43	7.60
N71Hie	7.28	7.63
N71F	7.41	7.64
N71W	7.52	7.71
N71Q	7.33	7.72

N71L	7.62	7.74
N71M	7.63	7.96
N71Y	7.99	8.22
N71K	9.05	9.55
N71Hip	9.23	9.59
N71R†	9.66	10.10
R73E	3.49	3.58
R73D	3.75	3.81
R73A	4.66	4.68
R73G	4.65	4.68
R73T	4.62	4.69
R73V	4.67	4.70
R73C	4.66	4.71
R73I	4.66	4.72
R73Hid	4.66	4.73
R73S	4.64	4.73
R73L	4.66	4.73
R73Q	4.66	4.75
R73M	4.66	4.75
R73N	4.69	4.77
R73F	4.74	4.80
R73Y	4.75	4.81
R73Hie	4.80	4.87
R73W	4.90	4.98
R73Hip	5.93	6.01
R73K	5.90	6.04
R73*	7.99	8.09
K84D	7.60	7.71
K84E	8.03	8.22
K84G	10.42	10.47
K84A	10.45	10.53
K84S	10.53	10.73
K84V	10.69	10.75
K84T	10.64	10.82
K84I	10.73	10.85
K84C	10.76	10.88
K84N	10.94	11.09
K84Y	10.91	11.15
K84L	11.16	11.29
K84Q	11.18	11.37
K84Hie	11.34	11.50
K84M	11.27	11.51

K84Hid	11.50	11.69
K84F	12.14	12.25
K84W	12.23	12.38
K84Hip	14.46	14.62
K84R	15.94	16.31
K84*	16.92	17.19
A85W	9.32	9.98
A85E	18.70	19.59
A85D	21.78	23.25
A85F	24.92	25.27
A85Q	24.66	25.85
A85Hie	25.56	26.48
A85Y	25.36	26.61
A85C	25.82	26.68
A85N	25.84	26.67
A85*	26.29	26.85
A85G	26.15	26.85
A85T	26.08	27.11
A85S	26.04	27.11
A85M	26.31	27.13
A85Hid	26.49	27.41
A85Hip	30.10	31.04
A85K†	30.67	32.30
A85R	31.44	32.69
K87E	11.72	11.94
K87D	11.96	12.20
K87G	12.61	12.74
K87A	12.70	12.83
K87Q	12.74	12.99
K87S	12.61	13.07
K87C	12.86	13.08
K87M	12.80	13.11
K87W	12.88	13.14
K87V	12.98	13.15
K87N	12.96	13.21
K87I	13.00	13.23
K87T	12.82	13.29
K87L	13.24	13.55
K87Hid	13.28	13.57
K87Hie	13.33	13.59
K87R	13.55	14.11
K87*	13.42	14.14

K87F	13.99	14.15
K87Y[†]	14.06	14.24
K87Hip	13.90	14.25
V88E	11.67	11.85
V88L	12.14	12.42
V88D	13.16	13.26
V88G	14.09	14.20
V88Q	14.76	14.92
V88A	14.98	15.09
V88S	15.02	15.18
V88M	15.32	15.59
V88C	15.61	15.73
V88I	15.68	15.84
V88N	15.81	15.99
V88T	15.93	16.07
V88[*]	16.46	16.61
V88Hid	16.65	16.81
V88K	16.56	16.94
V88Hie	16.83	16.96
V88Y[†]	17.34	17.55
V88Hip	17.65	17.83
V88R	17.52	17.95
V88F[†]	17.99	18.15
V88W	18.55	18.71
R89Y	−26.90	−26.05
R89F	−23.87	−22.50
R89L	10.43	11.19
R89D	11.43	11.88
R89E	12.74	13.18
R89V	13.77	14.23
R89Hid	13.61	14.40
R89G	14.22	14.46
R89A	14.52	14.78
R89T	13.94	14.81
R89S	14.53	15.00
R89N	14.66	15.14
R89C	15.00	15.27
R89Hie	14.66	15.37
R89I	15.20	15.57
R89Q	16.05	16.50
R89Hip	16.00	16.66
R89M	16.63	17.07

R89K	18.77	19.44
R89*	22.24	22.67

References

[1] A. A. Ojewole, J. D. Jou, V. G. Fowler, and B. R. Donald. *BBK* (branch and bound over K*): a provable and efficient ensemble-based algorithm to optimize stability and binding affinity over large sequence spaces*. In Springer International Publishing, 2017, pages 157–172.
