# Supplementary material for: Novel, provable algorithms for efficient ensemble-based computational protein design and their application to the redesign of the c-Raf-RBD:KRas protein-protein interface
Source: PLoS Comput Biol. 2020 Jun 8;16(6):e1007447. doi: 10.1371/journal.pcbi.1007447 (PMC7329130; doi:10.1371/journal.pcbi.1007447)
Supplement: S4 Table — For each listed variant, we give the dissociation constant Kd for each BLI titration experiment calculated from the fit done using the built-in mass transport model within the Octet Data Analysis HT software provided by FortéBio. We only accepted fits with a sum of square deviations χ2 less than 1 (FortéBio recommends a value less than 3) and a coefficient of determination R2 greater than 0.98. Presented in the table in Fig 10 are averages of these Kd values. (PDF) [file pcbi.1007447.s009.pdf]

Novel, provable algorithms for efficient ensemble-based computational protein design and their application to the redesign of the c-Raf-RBD:KRas protein-protein interface (Supporting information)
Anna U. Lowegard†, Marcel S. Frenkel†, Graham T. Holt, Jonathan D. Jou, Adegoke A. Ojewole, and Bruce R. Donald

† These authors contributed equally to the work.

S4 Table. K_d values for each tested variant for all replicates of BLI titration experiments.

For each listed variant, we give the dissociation constant K_d for each BLI titration experiment calculated from the fit done using the built-in mass transport model within the Octet Data Analysis HT software provided by ForteBio. We only accepted fits with a sum of square deviations χ^2 less than 1 (ForteBio recommends a value less than 3) and a coefficient of determination R^2 greater than 0.98. Presented in the table in Fig 10 are averages of these K_d values.

Run	Variant	K_d (M)	χ^2	R^2
1	c-Raf-RBD(RKY)	4.83×10^{-9}	0.0462	0.9997
2	c-Raf-RBD(RKY)	4.01×10^{-9}	0.0529	0.9997
3	c-Raf-RBD(RKY)	2.04×10^{-9}	0.211	0.9947
4	c-Raf-RBD(RKY)	2.15×10^{-9}	0.6266	0.997
1	c-Raf-RBD(RK)	1.47×10^{-8}	0.2232	0.9972
2	c-Raf-RBD(RK)	1.59×10^{-8}	0.2303	0.9975
3	c-Raf-RBD(RK)	1.58×10^{-8}	0.2269	0.9895
1	A85K	5.18×10^{-8}	0.3814	0.9957
2	A85K	4.53×10^{-8}	0.1414	0.9885
1	V88Y	3.86×10^{-8}	0.7742	0.9914
2	V88Y	2.59×10^{-8}	0.1658	0.9898
1	Wild-Type	1.31×10^{-7}	0.0971	0.9938
2	Wild-Type	1.01×10^{-7}	0.2561	0.9917
3	Wild-Type	1.17×10^{-7}	0.4417	0.9856
