# Supplementary material for: Novel, provable algorithms for efficient ensemble-based computational protein design and their application to the redesign of the c-Raf-RBD:KRas protein-protein interface
Source: PLoS Comput Biol. 2020 Jun 8;16(6):e1007447. doi: 10.1371/journal.pcbi.1007447 (PMC7329130; doi:10.1371/journal.pcbi.1007447)
Supplement: S1 Figure — c-Raf-RBD variants at 250 nM were allowed to associate with KRasGppNHp immobilized on a Ni-NTA OctetRed96 BLI tip for 180 s and then dissociation was measured and fitted for 120 s. All dissociation fits were performed in a local 1:1 model and showed strong agreement with the data, every fit having greater than a R2 of 0.99 and a χ2 lower than 0.01. Each curve is labeled with its corresponding c-Raf-RBD variant boxed in the matching color. A triplicate repeat was performed for the c-Raf-RBD wild-type (WT) variant (Red). Curves grouped into three groups: variants similar to WT (T57K in blue, T57M in cyan, WT in red, K87Y in orange, and V88F in forest green), variants better than WT (A85K in pink, N71R in sand, and V88Y in black), and variants with a response greater than twice that of the WT (RK in purple and RKY in green). (PDF) [file pcbi.1007447.s010.pdf]

Novel, provable algorithms for efficient ensemble-based computational protein design and their application to the redesign of the c-Raf-RBD:KRas protein-protein interface (Supporting information)

Anna U. Lowegard†, Marcel S. Frenkel†, Graham T. Holt, Jonathan D. Jou, Adegoke A. Ojewole, and Bruce R. Donald

† These authors contributed equally to the work.

S1 Fig. Curves for single concentration BLI screen of c-Raf-RBD variants.

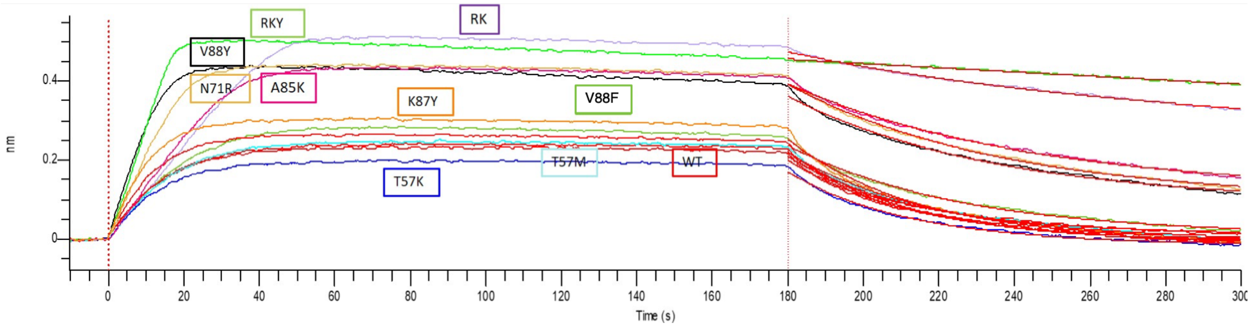

c-Raf-RBD variants at 250 nM were allowed to associate with KRas^{GppNHp} immobilized on a Ni-NTA OctetRed96 BLI tip for 180 s and then dissociation was measured and fitted for 120 s. All dissociation fits were performed in a local 1:1 model and showed strong agreement with the data, every fit having greater than a R^2 of 0.99 and a χ^2 lower than 0.01. Each curve is labeled with its corresponding c-Raf-RBD variant boxed in the matching color. A triplicate repeat was performed for the c-Raf-RBD wild-type (WT) variant (Red). Curves grouped into three groups: variants similar to WT (T57K in blue, T57M in cyan, WT in red, K87Y in orange, and V88F in forest green), variants better than WT (A85K in pink, N71R in sand, and V88Y in black), and variants with a response greater than twice that of the WT (RK in purple and RKY in green).
