# Supplementary material for: Novel, provable algorithms for efficient ensemble-based computational protein design and their application to the redesign of the c-Raf-RBD:KRas protein-protein interface
Source: PLoS Comput Biol. 2020 Jun 8;16(6):e1007447. doi: 10.1371/journal.pcbi.1007447 (PMC7329130; doi:10.1371/journal.pcbi.1007447)
Supplement: S2 Figure — Titration experiments were conducted over different concentration ranges and for different association and dissociation times in order to avoid artifacts. Within each titration experiment, curves were fit globally to a mass transport model using the FortéBio Data Analysis HT software. All fits achieved an R2 greater than 0.99 and a χ2 smaller than 0.65. The two titration experiments on the left are replicates with concentrations ranging from 150 nM to 4.69 nM in a 2-fold serial dilution. The titration experiment on the top right has titrations ranging from 150 nM to 9.38 nM in a 2-fold serial dilution but with an extended association step. The titration in the bottom right contains binding curves with the following concentrations of c-Raf-RBD(RKY): 200 nM, 125 nM, 75 nM, 75 nM, 25 nM, 25 nM, and 10 nM. Note the in-experiment repetition of two concentrations (75 nM and 25 nM). This was done in order to control for response and curve shape within an experiment. Curves for the repeat concentrations show strong reproducibility and alternating what repeat curves are used for the global fit changes the Kd within a range of 1.99 nM to 2.34 nM. Results from these four titration experiments were averaged to generate a dissociation constant and standard deviation for c-Raf-RBD(RKY). Results are reported in the manuscript as the dissociation constant ± two standard deviations. (PDF) [file pcbi.1007447.s011.pdf]

Novel, provable algorithms for efficient ensemble-based computational protein design and their application to the redesign of the c-Raf-RBD:KRas protein-protein interface (Supporting information)

Anna U. Lowegard†, Marcel S. Frenkel†, Graham T. Holt, Jonathan D. Jou, Adegoke A. Ojewole, and Bruce R. Donald

† These authors contributed equally to the work.

S2 Fig. Replicate BLI titration curves of c-Raf-RBD(RKY) binding to immobilized KRas on NiNTA tips.

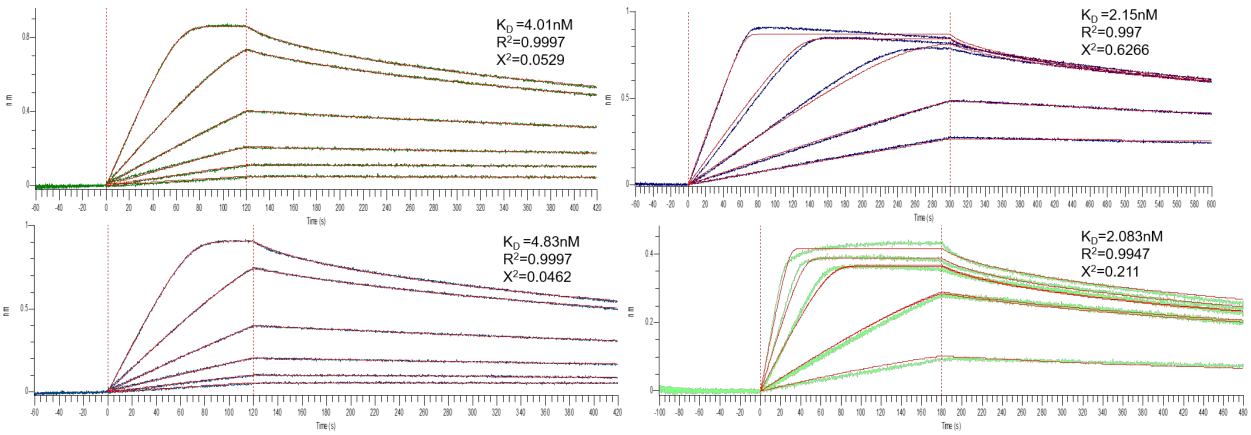

Titration experiments were conducted over different concentration ranges and for different association and dissociation times in order to avoid artifacts. Within each titration experiment, curves were fit globally to a mass transport model using the FortéBio Data Analysis HT software. All fits achieved an R^2 greater than 0.99 and a χ^2 smaller than 0.65. The two titration experiments on the left are replicates with concentrations ranging from 150 nM to 4.69 nM in a 2-fold serial dilution. The titration experiment on the top right has titrations ranging from 150 nM to 9.38 nM in a 2-fold serial dilution but with an extended association step. The titration in the bottom right contains binding curves with the following concentrations of c-Raf-RBD(RKY): 200 nM, 125 nM, 75 nM, 75 nM, 25 nM, 25 nM, and 10 nM. Note the in-experiment repetition of two concentrations (75 nM and 25 nM). This was done in order to control for response and curve shape within an experiment. Curves for the repeat concentrations show strong reproducibility and alternating what repeat curves are used for the global fit changes the K_d within a range of 1.99 nM to 2.34 nM. Results from these four titration experiments were averaged to generate a dissociation constant and standard deviation for c-Raf-RBD(RKY). Results are reported in the manuscript as the dissociation constant $\pm$ two standard deviations.
