# Supplementary material for: Novel, provable algorithms for efficient ensemble-based computational protein design and their application to the redesign of the c-Raf-RBD:KRas protein-protein interface
Source: PLoS Comput Biol. 2020 Jun 8;16(6):e1007447. doi: 10.1371/journal.pcbi.1007447 (PMC7329130; doi:10.1371/journal.pcbi.1007447)
Supplement: Attachment — Submitted filename: RTR.pdf [file pcbi.1007447.s013.pdf]

# How we responded to reviewers' comments: Novel, Provable Algorithms for Efficient Ensemble-Based Computational Protein Design and Their Application to the Redesign of the c-Raf-RBD:KRas Protein-Protein Interface

Anna U. Lowegard    Marcel S. Frenkel    Graham T. Holt
Jonathan D. Jou    Adegoke A. Ojewole    Bruce R. Donald

We found the reviewers' comments very helpful, and we thank them for their suggestions which have greatly improved our manuscript. Below, we address each comment individually and summarize the resulting changes to the manuscript. While changes to the manuscript have been made throughout, the major ones are indicated in red. References (e.g., [1]) can be found at the end of this response. Added bibliographic references are not in red in the manuscript due to limitations of LaTex.

In response to Reviewer 1's questions about the significance of our designed c-Raf-RBD(RKY) variant, we attempt to answer these questions in this response to reviewers. However, we do agree to tone down our claims of significance, as she/he suggested, and have done so in the revised manuscript. We agree it is best to present our data, suggest the criteria to evaluate it, and let the reader decide. For this reason, our answers herein to Reviewer 1 are designed to explain our excitement about and interpretation of our data. But, conceding to the reviewer's preference, the arguably over-enthusiastic language of multiple published papers in the Ras field (for example, characterizing previous 5- to 7- fold binding improvements as "superbinders" — their term — a phenomenon we discuss below on pages 10-11) is not propagated to the revised manuscript. We do feel that the intense previous search for mutations to improve Ras:Raf binding was motivated by the biomedical significance of understanding this PPI. Moreover, it is surprising to us that this search, spanning multiple papers and techniques (ranging from experimental to computational), did not discover our designed variant. This suggests that it was not an easy mutant to find, despite a great deal of previous research. Altogether, we feel this background literature strengthens the case for publication of our manuscript, since it documents that affinity improvements in this 5-7 fold range have been deemed significant previously, by sound papers in good journals. By presenting this literature together with our data, while not insisting on this interpretation in the revised manuscript itself, we hope the reviewers will now feel that our revised paper is responsive to their comments. Finally, we appreciate this opportunity to engage in a discussion with reviewers and editors, and to present the evidence, as we see it, for how to frame our results. Thank you.

# Reviewer 1

Lowegard et al. present a development of the Donald lab Osprey design methodology to improve efficiency in the design of large sequence ensembles. If I understood correctly, Osprey carries out a combined sequence-design and backbone relaxation step and therefore requires enumerating sequence space. To reduce the enumerated space to a size that can be practically computed, they now compute the stability of each component of the protein system (for instance, receptor and ligand) to ensure that no mutant is too destabilising before considering all combined mutations. The authors tested their approach on a natural (and engineered) PPI involving KRas and retrospectively tested the ranking of mutants relative to previous mutational analyses. They also used the method to design a single-point mutant and found that it improved affinity five-fold relative to the starting point.

***Answer:***

1. While OSPREY is capable of designing with backbone movements [21, 22], it does not carry out combined sequence-design and backbone relaxation steps. Any provable protein design algorithm must search (but not necessarily enumerate) the entire sequence space for a design – this is not a consequence of backbone flexibility but rather a requirement for a provable algorithm. In other words, the power of these algorithms is that every sequence is either enumerated or pruned, and a combinatorial number of sequences can be pruned without being enumerated. The result is that no sequence is missed, and the optimal sequences (relative to the input model) are returned. Please see the response to reviewer 2 on pages 19-20 for more detail on this point.

2. We tested this work not only on the KRas:c-Raf-RBD PPI both prospectively and retrospectively, but also on designs selected from 40 different protein-protein interfaces (See the sections labeled "Computational experiments" and "Computational results" along with the list of systems in Table S2). FRIES alone was tested on 2,662 designs and FRIES/$EWAK^*$ in combination were tested on 167 design examples. These experiments show that FRIES/$EWAK^*$ are more efficient than the previous state-of-the-art approach, $BBK^*$. We have emphasized this more throughout the revised manuscript and, in particular, we added the following to the abstract:

   > "This combined approach led to significant speed-ups compared to the previous state-of-the-art multi-sequence algorithm, $BBK^*$, while maintaining its efficiency and accuracy, which we show across 40 different protein systems and a total of 2,826 protein design problems."

3. Finally, the resulting variant c-Raf-RBD binds 36 times more tightly to KRas than the design starting point and 5 times more tightly than the previous tightest-known binder. We have revised the manuscript to more clearly highlight these points. To emphasize this we added the following to the abstract:

> "This new variant binds roughly five times more tightly than the previous best known binder and roughly 36 times more tightly than the design starting point."

> The main message of the paper is rather difficult to distill. I wrote above how I understood the motivation for the current paper and how the authors addressed the computational problem, but this not stated in the abstract and it was only by going through the methods that I could understand this (and I'm still not sure that I understood correctly). The methods are, as expected for such a paper, quite dense with formulae so the big picture is lost on a reader. My first suggestion is to clarify already in the abstract what is the main contribution of this paper, not just by stating that the method is more efficient, but why it is more efficient and for which problems. In this connection, the provability of the method is of far less significance to most users and developers of computational design methodology than its practical usefulness (accuracy, speed, scope). Nevertheless, the point about provability is repeated from the second word of the title to the end of the paper in the excess of 30 times. My second suggestion is therefore to substantially reduce the emphasis from this point and highlight applicability and accuracy.

***Answer:***
We appreciate this feedback, and have clarified the main message of the paper both in the abstract and more generally. Additionally, we have reduced the emphasis on provability to only where we feel it is of crucial importance and have highlighted the applicability of the methods presented in the manuscript. In particular, the reviewer's point about the abstract has been addressed by adding the following:

> "FRIES pre-processes the sequence space to limit a design to only the most stable, energetically favorable sequence possibilities. $EWAK^*$ then takes this pruned sequence space as input and, using a user-specified energy window, calculates $K^*$ scores using the lowest energy conformations. We expect FRIES/FRIES to be most useful in cases where there are many unstable sequences in the design sequence space and when users are satisfied with enumerating the low-energy ensemble of conformations. In combination, these algorithms provably retain calculational accuracy while limiting the input sequence space and the conformations included in each partition function calculation to only the most energetically favorable, effectively reducing runtime while still enriching for desirable sequences. This combined approach led to significant speed-ups compared to the previous state-of-the-art multi-sequence algorithm, $BBK^*$, while maintaining its efficiency and accuracy, which we show across 40 different protein systems and a total of 2,826 protein design problems."

To further emphasize this point we have also added the following to the manuscript in the Section labeled "Background":

"These previous methods, while efficient, suffer from two practical drawbacks. First, some returned sequences exhibit a large $K^*$ score due to a decrease in stability of the unbound states. These sequences are rarely desirable in practice, since decreasing protein stability can result in poor folding and aggregation. Second, the approximation error for some sequences is slow to approach epsilon which can lead to prohibitively slow designs."

We also added the following to the Section labeled "Energy window approximation to K* ($EWAK^*$)":

"Limiting the partition functions to only these energetically favorable conformations can effectively reduce runtime while still enriching for desirable sequences."

The authors chose the KRas system apparently because it is a drug target. They mention several times that KRas is undruggable and that this is therefore a biomedically significant problem. First, as the authors mention at one point in the text, KRas is actually not undruggable and there are now small molecules in clinical trials (or in the clinic; I'm not sure). Second, whether or not KRas is a drug target is beside the point for this paper, since KRas is an intracellular target and no matter what affinities the authors achieved, the designs could not be used in any conceivable way in treatments or even in drug discovery. I therefore recommend that they drop all reference to druggability and concentrate on the truly important aspect, which is that KRas has been studied extensively as a model PPI, thus providing an excellent data set for retrospective analysis.

***Answer:***

We have removed the phrase "undruggable" from the manuscript and replaced it with more precise language. Overall, as suggested by reviewer 1, we have shifted focus toward the c-Raf-RBD:KRas protein-protein interface as a model system, rather than a drug target. In particular, we made large changes to the Section labeled "Computational redesign of the c-Raf-RBD:KRas" in regards to the motivation behind selecting this system. However, we believe that any insights into the PPI mechanism of Ras:Raf recognition through improved mutations is potentially relevant to biomedical understanding, given the intense search for these improved binders previously [2,4–6,8,10–12,18,19,24,26,27,31–33,35,42,45–47]. Moreover, all previous improved binders supercharged RBD, limiting their significance, whereas ours make aromatic substitutions. Finally, while the particular c-Raf-RBD variant presented herein is not being considered as a drug candidate, simply attaching this design to a cell-penetrating peptide (e.g., 8R) could allow disruption of effector signalling in cell culture, and is therefore a potential reagent for further characterizing the *in vitro* signalling of KRas. We submit that it is indeed appropriate to describe such a tool as biomedically relevant, since such a reagent could enable new experiments and better characterization of the interaction between KRas and its effectors, a system that has been extensively studied for purposes of drug discovery [2,4–6,8,10–12,18,19,24,26,27,31–33,35,42,45–47]. We have added sentences to this effect in the Discussion.

The narrative of this study is confusing: the authors developed new methods to enable design within large sequence spaces, but in the end, they validated their method by testing it retrospectively against a predetermined set of mutants and in the prospective design of a single-point mutation. It seems that any molecular threading approach and the simplest mutational scanning method (FOLDX?) would be just as useful for this analysis as the newly developed method. This is, in my opinion, a critical point. I don't think that the validation presented here is sufficient and instead, the authors should show that in the design of large ensembles, they recapitulate known sequence signatures (for instance, natural sequence alignments), and they should do this for more than one protein. Such a benchmark would show that the method is indeed fast enough to be practically useful for large sequence spaces and that it yields more than anecdotal successes.

***Answer:***

The reviewer raises two main points in this comment that we will address separately:

1. The reviewer argues that validation of the accuracy of this method has not been performed adequately.
2. The reviewer argues that validation of the speed of this method has not been performed adequately.

Our response follows:

1. One of the advantages of provable design algorithms is that they are guaranteed to return the optimal result (within user specified error) for a given input model. This is in sharp contrast to stochastic methods, which frequently return different results depending on a variety of factors, including initial state. The experimental accuracy of the $K^*$ design algorithm has been validated extensively against experimental data on a variety of systems, including HIV bNAbs, inhibitors for cystic fibrosis, and dihydrofolate reductase, among others [7, 9, 13, 16, 37–39, 43] using experimental measurements of binding, crystal structures, NMR structures, and in-cell measurements.

   For this reason, in addition to testing the accuracy of $EWAK^*$ and FRIES both retrospectively and prospectively on the KRas:RBD interface, we performed 2,826 designs across 40 different protein-protein interfaces and compared the results to those returned by $BBK^*$, which also enjoys the provable guarantees of $K^*$. In addition to achieving excellent prediction accuracy for the KRas:RBD system (See the section entitled "FRIES/ $EWAK^*$ retrospectively predicted the effect of mutations in c-Raf-RBD," FRIES and $EWAK^*$ achieved excellent concordance with $BBK^*$ on 40 protein-protein interfaces. Therefore, we argue that our new algorithms are of comparable accuracy with previous provable methods. We have highlighted this point by adding the following to the section titled "EWAK* limits the number of minimized conformations when approximating partition functions while maintaining accurate K* scores" in the revised manuscript:

"This indicates that *EWAK** retains accuracy when compared to previous provable algorithms, which have been extensively validated using experimental measurements of binding, crystal structures, and NMR structures on a variety of systems [7,9,13,16,37–39,43]. The accuracy of *EWAK** is explored further in Section 5.1, where we perform additional retrospective validation against experimental measurements."

Furthermore, simple mutational scanning methods like ORBIT [8] appear to be less useful than our method in at least one case, evidenced by their inability to predict the improved RBD variant presented in this manuscript. It has also been shown in [41] that Rosetta, a widely-used heuristic sampling method, is unlikely (and possibly unable) to efficiently find the optimal solution of a design problem. Additionally, although FOLDX has been shown to correlate well with experimental measures of binding for point mutations [3, 28], that correlation can vanish when considering more than one mutation site [48]. In contrast, our method maintains accuracy when considering double and triple mutations (See Figure 5). Furthermore, FOLDX has previously been applied to the KRas/RBD interface [28] but did not discover the c-Raf-RBD(RKY) variant reported herein, suggesting that our methods do in fact provide utility relative to FOLDX. Finally, the ability to perform native sequence recovery does not guarantee the ability to predict mutations that improve binding. For design of kCAL01 [38] and VRC07-523LS [39], and resistance prediction in DHFR [9, 37], non-native sequences were required and were found by OSPREY. These predictions were validated not only biochemically and structurally, but also at an organismal level, in animals, and in humans.

In sum, we believe not only that the most useful validation of algorithm accuracy should be a comparison based on experimental measurements and first principles, but also that this validation has already been done.

2. In order to test the ability of FRIES and *EWAK** to design on large sequence spaces we compared the runtime of our new algorithms against the runtime of $BBK^*$ for 2,826 designs across 40 different protein-protein interfaces, with a maximum sequence space size of 9261. We showed that FRIES and *EWAK** ran up to two orders of magnitude faster, and reduced the sequence space by more than two orders of magnitude in the best case. We submit that this validation is sufficient to show that our new algorithms are able to perform more efficiently than the previous state of the art on large sequence spaces. We have emphasized this more throughout the revised manuscript and, in particular, we added the following to the abstract:

   "This combined approach led to significant speed-ups compared to the previous state-of-the-art multi-sequence algorithm, $BBK^*$, while maintaining its efficiency and accuracy, which we show across 40 different protein systems and a total of 2,826 protein design problems."

Our algorithms have been extensively validated against experimental data [7, 9, 13, 16, 37–39, 43]. Because our methods are provable, this validation also applies to FRIES and

$EWAK^*$. This is because provable algorithms are guaranteed to return the same result. Moreover, we validated the accuracy of FRIES and $EWAK^*$ on 2,826 designs. Finally, we believe that our results are sufficient to show that FRIES and $EWAK^*$ are more efficient than the previous state of the art on large sequence spaces. To further emphasize this and the real-world application of the algorithms we have added the following to the Section labeled "FRIES/$EWAK^*$ retrospectively predicted the effect mutations in c-Raf-RBD have on binding to KRas" in the manuscript:

> "These cases in particular serve as examples of large designs where $EWAK^*$ outperforms $BBK^*$ and highlight the utility of FRIES/$EWAK^*$ when considering larger designs."

> Regarding the single-point mutant that the method prospectively designed. This mutant showed at most five-fold improvement in affinity over the starting point engineered variant. This is quite modest to say the least, though the authors trumpet this result as "a discovery of some significance." The tone relating to this mutant should be reduced throughout the paper.

***Answer:***

We have moderated the tone regarding the designed KRas mutant throughout the paper. However, we believe that it would be overly cautious to describe a 5-fold improvement in affinity as "quite modest to say the least."

In a biological system, protein binding and specificity results from complex interactions involving multiple partners, and is therefore context-dependent [40]. Therefore we do not find it obvious that the 5-fold improvement in affinity of a c-Raf-RBD variant previously described as a "superbinder" [8] would result in only modest effects. We have refrained from using the term "superbinder" to describe our designed construct, but note that experts in the Ras:Raf field have used this term for comparable improvements in affinity. We submit that the improvement in affinity of 5X is potentially significant, especially since this represents a 36X improvement in affinity over wild-type.

Finally, we present two cases [25, 38, 39] in which improvements in binding of 3-7X were biologically and physiologically significant. Both of these designs used OSPREY/$K^*$, and here we believe the comparison will be useful. We are authors on these previous studies. Consider the case of the anti-HIV broadly-neutralizing antibody (bNAb) VRC07. Improvements to VRC07 reported in [39] resulted in the antibody VRC07-523LS, which exhibited only a 3.4-fold improvement over the original VRC07 antibody (calculated using geometric mean of IC50 over a panel of 179 HIV-1 Envelope pseudoviruses). Similarly, the $K_d$ was improved by approximately 5X. First, these improvements in affinity resulted in similar (5-8X) improvement in *in vivo* potency in monkeys [39] for VRC07-523LS. Second, only one clinical trial of VRC07 has been reported (ClinicalTrials.gov identifier: NCT03374202), but 13 clinical trials are listed for VRC07-523LS at the time of writing. These trials include 3 phase II, and very promising results have already been reported in *The Lancet* [1]. Thus, an apparently modest improvement in binding resulted in significantly greater clinical impact. Similarly, with

kCAL01 [38], a drug lead for CFTR, OSPREY/$K^*$ improved binding by roughly 7-fold. This improvement was in part due to successful modeling of entropic effects by OSPREY [25], and resulted in a significant improvement in chloride ion transport in human cell-based models of cystic fibrosis. We showed that, since they lacked the 7X improvement in $K_d$ obtained by the OSPREY/$K^*$ designs, previous inhibitors lacked the *in vivo* efficacy of kCAL01.

> The BLI experimental methods are described far too briefly to understand what was actually done and what fits are reported. It is not clear what model was used to fit the data. It looks to me like the authors may have fitted a kinetic model but I'm not sure whether they fitted each curve to a separate kinetic model or all curves at once (the latter is the correct way of analysing these data). The fits to the RK variant (Fig10) are quite poor. Considering that they have 6 independent measurements, and 4 of them show poor fits, I think that this experiment needs to be redone and since this serves as the baseline measurement to judge the impact of the V–¿Y designed mutation, this is quite important. I also suggest to draw the fits in black because some of the traces are quite close in tone to the red used to show the fits.

***Answer:*** We thank the reviewers for these comments, and have added the new SI figures (S2 and S3) to address them. In addition, the BLI methods sections have been re-written to reflect the concerns of the reviewer. In the revised manuscript we clarify that experiments have been done in triplicate for c-Raf-RBD(RK) and c-Raf-RBD(RKY) (see newly added Figs S2 and S3). Moreover, the model used for fitting has been explained as has the fact that curves were globally fit (we agree with the reviewer that this is the proper fitting). More detail has been added to the Section labeled "Experimental validation of mutations in the c-Raf-RBD:KRas protein-protein interface" to hopefully sufficiently describe the experiments. Additionally, the fit is well within what is recommended by the manufacturer for reporting [44]. We would also like to note that the $K_d$ we determined and reported for the c-Raf-RBD(RK) variant is nearly identical to that reported in the literature [8] which gives us even more confidence in the accuracy of the $K_d$ values we report in the manuscript.

> Also, typically when such modest improvements in affinity are reported (five-fold), and given that the fits are rather poor in the data that were presented, it's important to provide experimental replicates. It is difficult to say whether the five-fold effect is real or within the noise of the experimental setup.

***Answer:***

We do have experimental replicates that we give data for in the SI, but we did not previously provide plots. We have updated this and added the requested plots to the SI for the important c-Raf-RBD variants, c-Raf-RBD(RKY) and c-Raf-RBD(RK). These two variants are our new designed variant and the previous tightest-known binding variant, respectively. Please see Figs S2 and S3.

Eq. 2: define C,P,L

***Answer:***

C,P, and L are defined, but not right next to equation 2. This has been updated and made more clear in the revised manuscript by adding the following just above Equation 2:

> "...where $C$, $P$, and $L$ refer to the protein-ligand complex, the unbound protein, and the unbound ligand, respectively"

2. On pg. 17, the authors use Spearman correlations to check the rank order correlation in 38 mutations. Since the computational method reports energies and the binding experiments report KDs, why not use Pearson correlations?

***Answer:***

Briefly, our current designs likely underestimate entropic contributions to binding, as they only model a subset of biologically available flexibility and do not model explicit solvent molecules. Furthermore, most physical effective energy functions (including the default used in OSPREY) are based on small-molecule energetics, which can overestimate van der Waals terms and thereby overestimate internal energy. To clarify this we have added the following to the manuscript at the end of the Section labeled "FRIES/$EWAK^*$ retrospectively predicted the effect mutations in c-Raf-RBD have on binding to KRas":

> "We use Spearman's $\rho$ here as opposed to a Pearson's correlation since our current designs likely underestimate entropic contributions to binding due to various limitations in biological modeling. Despite these model limitations, in [23, 37] large changes in $K^*$ score corresponded to significant changes in energy, and rankings correlated well with experimental binding measurements. The Spearman's $\rho$ for the study presented here is comparable to the values for other PPI systems when using OSPREY [23, 37]. Furthermore, an accurate ranking can guide an experimental lab in choosing the rank order in which to test computational predictions [7, 9, 13–17, 30, 34, 37–39, 43]"

Line 403: the authors state that they filtered mutations based on "promising K* scores and structure examination." It's important that they provide some guidelines about how they selected the mutation. This explanation cannot be reproduced by anyone.

***Answer:***

We added an explanation of mutation filtering to the end of the section titled: "Prospective redesign of the c-Raf-RBD:KRas protein-protein interface toward improve binding." The following has been added to the manuscript to clarify:

"Of the mutations selected, T57M was selected to act as a variant that we computationally predicted to be comparable to wild-type. This variant was included to further verify the accuracy of OSPREY's predictions. On the other hand, some of OSPREY's top predictions were excluded, for instance, T57R (included in S3 Table) was not selected for experimental testing because it has an unsatisfied hydrogen bond as evidenced in the structures calculated by OSPREY. Another example is position V69 where 3 different mutations are predicted to improve binding, however, this position was included in our retrospective study (see Section labeled "FRIES/$EWAK^*$ retrospectively predicted the effect mutations in c-Raf-RBD have on binding to KRas" and Fig 5) and was 1 of only 3 positions where OSPREY incorrectly predicted the effect of the mutation. Therefore, we do not believe that the scores accurately represent the effect the mutations will have in these few cases. Other excluded top predictions (see S3 Table) displayed unsatisfied hydrogen bonds or have been reported and tested previously [8,10,27]. One special case that is not shown in our experimental validation below is V88W which caused poor expression of c-Raf-RBD so we were unable to test it."

The single-concentration BLI measurements are very unusual (Fig8). I'm not sure that they are meaningful at all since at a single concentration it's impossible to determine $K_d$ and it's not very obvious what is the signal one measures. I recommend to drop this analysis as it is misleading, especially since the authors draw conclusions on the correlation between these experimental results and the computational analysis.

***Answer:***

We thank the reviewers for their insight. The concerns are well taken and to accommodate the reviewer we no longer approximate binding affinity using data from this assay. We have recreated the figure to focus on response and dissociation rates. Because the c-Raf-RBD variants are of similar size and are all screened at the same concentration, the response rate and dissociation rate can be used for screening purposes, as is well established previously in Refs: [29, 36, 49]. It is important to note that this method is used merely as a screen to filter and pick which mutations to subject to further experimental validation. It is also worth noting that the results of the subsequent titration experiments align excellently with the results of this screen (see Figs 8 and 10). Additionally, we have modified sections "Prospective redesign of the c-Raf-RBD:KRas protein-protein interface toward improved binding" and "Experimental validation of mutations in the c-Raf-RBD:KRas protein-protein interface" to reflect this.

Lines 433-437: The authors make a lot of the five-fold improvement that the single point mutation exhibited. It's a quite modest improvement and seems almost anecdotal given that no other mutants were presented.

***Answer:***

We have moderated the language in this passage. Please see pages 10-11 above for a discussion on the relevance of a five-fold improvement in binding affinity.

We believe a strength of our method is that only a few mutants need to be created and tested to discover a potentially significant increase in binding. Competing methods might require screening for more mutants, and the benefit of computation is that it can reduce the need for extensive screening when an improvement in binding is desired.

Lines 439-452: this belongs in methods.

***Answer:***

A section heading entitled "Experimental materials and methods" has been added to clarify and delineate the methods from the other sections.

475: "we applied these algorithms to a biomedically significant design problem": this is not a biomedically significant design problem, because the design cannot be used in a biomedical context. KRas is biomedically significant drug target but the design is in no way a starting point for a drug. Again, the authors can highlight the datasets that are available for KRas but not the druggability aspects as they have no relevance to the work.

502: "the discovery that such a mutation can improve the binding...is of considerable significance...eventually developing successful therapeutics". This is quite spurious. They tested a single mutation which exhibited a very modest effect and has no relevance to drug discovery.

***Answer:*** We have moderated the tone in the indicated passages, and highlighted the available datasets for KRas. Please see pages 6-7 above for a discussion of the biomedical relevance of the RBD construct, and pages 10-11 above for a discussion of the predicted effects of a 5-fold improvement in binding affinity.

We screened 7 new variants (c-Raf-RBD(RKY), V88Y a.k.a. c-Raf-RBD(Y), N71R, V88F, T57M, K87Y, and T57K) and tested 2 novel variants, c-Raf-RBD(Y) and c-Raf-RBD(RKY), in detail. Additionally, we also screened and tested in detail 3 other variants: the wild-type c-Raf-RBD, c-Raf-RBD(RK), and A85K, which have been tested in previous studies [8]. We found one improved binder, which is significant because the total number of mutants we tested experimentally is small. If we had tested more mutations, it would, in fact, be less surprising to find an improvement. Furthermore, this interface has been extensively studied [2,4–6,8,10–12,18,19,24,26,27,31–33,35,42,45–47] and is considered highly optimized. In this light, is it not surprising to find any improvement, given the intense previous scrutiny and relatively small number of mutations tested in this manuscript?

# Reviewer 2

> The authors present two algorithmic improvements for the $K^*$ algorithm for predicting binding affinities at protein/protein interfaces, and then show that their improved algorithm is capable of accurately predicting binding energies with a retrospective analysis of mutations at the cRaf-RBD / KRas interface and by predicting several point mutations that improve binding relative to wild-type, including one mutant, V88Y, that when paired with two previously-reported mutations, creates the tightest-yet-known interface between these two proteins. The paper spans from theory to the bench and is an impressive piece of work.

***Answer:*** We thank the reviewer for the interest and positive evaluation of our work.

> The two algorithmic improvements the authors describe focus on the two levels at which the $K^*$ algorithm broaches an exponential amount of work, 1) at the conformation level, and 2) at the sequence level.
>
> At the conformation level, the $K^*$ algorithm is designed to approximate $K = q_{pl}/q_p q_l$ by enumerating all conformations of a single sequence in order of increasing energy until the conformations remaining are at a high-enough energy that their contribution to the partition function is small – small enough that the resulting $K^*$ approximation is within a user-provided constant, epsilon, of the full K. To improve performance here, the authors introduce the *EWAK** algorithm. *EWAK** instead of enumerating conformations until the epsilon-error threshold is reached, they stop when the bound on the energy of the next conformation is larger than some user-provided deltaE of the best conformation. The authors contrast *EWAK** with the previous algorithm, *BBK**, and note that it produces significant performance improvements.
>
> I found this section a little difficult to understand: A) The algorithmic improvement presented in the *BBK** paper from 2017 focuses entirely on sequence-space pruning and not conformation-space pruning, and so *EWAK** seems like it ought to be compared to $K^*$ (or that the text should say that *BBK** and $K^*$ are the same for the sake of this comparison). B) It would be nice to understand how the user providing an energy threshold "1 kcal/mol" differs from the user changing epsilon to ".05" from "0.01". Is there a simple mathematical relationship between these numbers or are they related but not comparable?

***Answer:***

A) We have added language clarifying the relationship between the $K^*$ and $BBK^*$ algorithms to the introduction and a more detailed discussion to the SI. Briefly, $BBK^*$ calls a partition function approximation algorithm as a subroutine. In the submitted manuscript, we compare the previous partition function approximation algorithm ($K^*$) with the new one ($EWAK^*$).

B) The reviewer is correct in noting that the energy threshold and epsilon values are related, since increasing the energy threshold must decrease the value of epsilon, and vice

versa. Unfortunately, the precise function relating these two values is not entirely simple to calculate. We have added a discussion of this to the SI, and have added a sentence to the end of the section "Energy Window Approximation to $K^*$ ($EWAK^*$)" pointing the interested reader to this discussion.

> At the sequence level, the authors present an algorithm, FRIES, for removing sequences that are higher in energy than the wild type sequence. Here both FRIES and $BBK^*$ enumerate sequences in order of decreasing bounds on their energies. With FRIES, the intuition on when to stop is that, after the wild-type sequence is encountered at an interface where one is looking for tighter binding, then there is not much point in continuing.
>
> I found this section slightly confusing because it's not clear whether the energies are for the complex structure, the unbound structure, or both. It's also unclear why FRIES searches the multi-sequence tree until the wild type sequence is hit instead of going directly to the (clearly known!) wild type sequence. I believe what the authors mean to describe is that FRIES searches the multi-sequence tree and descends into the single-sequence conformation tree for each sequence it encounters until it hits the wild type sequence, after which point it begins looking to stop sequence enumeration.

***Answer:***

To clarify, $BBK^*$ does not have a comparable sequence filtering process. $BBK^*$ performs its search on the set of all conformations (which contains all possible sequences) to approximate $K^*$ scores. On the other hand, FRIES serves as a pre-process filter that limits the number of sequences that then go on to be included in the $K^*$ score approximations performed by $EWAK^*$. FRIES does 3 separate searches, one for the complex, one for the protein, and one for the ligand. The sequences that remain in the intersection of all three filtered sets are the ones that are kept and further processed by $EWAK^*$. This has been clarified in the Section labeled "Fast Removal of Inadequately Energied Sequences (FRIES)" with the following additional text:

> "The following algorithm is applied to each of the three states (protein, ligand, and protein-ligand complex) independently. The resulting, filtered sequence space is determined by taking the intersection of the output from the algorithm for the three states."

The purpose of searching until finding the wild-type sequence is that it allows FRIES to collect all of the sequences that have potentially better, lower energies. The energy that FRIES enumerates by is the lower-bound on the minimized energy for each sequence as described in [20]. Hence, FRIES enumerates up to the wild-type sequence and, as it does so, enumerates and keeps low-energy sequences. {Every sequence is either enumerated or provably pruned. If we merely calculated the energy of the wild-type sequence then we would not know the identity of the other low-energy sequences in the tree. Additionally, FRIES only descends into the single-sequence conformation tree (as described in the Section labeled "Fast Removal of

Inadequately Energied Sequences (FRIES)") for the wild-type sequence (it does not search this conformation tree for any other sequence) in order to find the energy of one specific, low-energy conformation of the wild-type sequence to provably bound the energies of the remaining sequences to be enumerated as described in the section labeled "Fast Removal of Inadequately Energied Sequences (FRIES)." To clarify this, we have added the following text to the Section labeled "Fast Removal of Inadequately Energied Sequences (FRIES)":

> "It is worth noting that FRIES only descends into and searches the single-sequence conformation tree for the wild-type sequence in order to calculate the provable halting criteria for Eq (3)."

> I would also be curious to understand why the authors chose to approximate $q_{wt}$ using only a single conformation of the wild-type sequence instead of trying to estimate $q_{wt}$ accurately; the wild type sequence is just one more sequence among the very many sequences that FRIES and *BBK*$^*$ would enumerate.

***Answer:*** We thank the reviewer for their curiosity and interest in the algorithms. For the purposes of FRIES, which is meant to be a very fast way of identifying low-energy sequences, it is not necessary to more accurately approximate the partition function for $q_{wt}$ since it is used merely as a lower bound to establish our halting criteria for the sequence search. Estimating the partition function using a single low-energy conformation allows us to put a lower-bound on the partition function for the wild-type sequence so we can set a cut-off for the partition functions of the other low-energy sequences (see Equations 5 and 6 and accompanying text in the Section labeled "Fast Removal of Inadequately Energied Sequences (FRIES)"). Once these sequences are identified, $EWAK^*$ is used to more accurately predict the values of the partition functions for each sequence including the wild-type sequence.

> Minor point: on page 5 the phrase "by up to more than 2 orders of magnitude" confuses me; is it "by up to 2 orders of magnitude" or "more than 2 orders of magnitude"?

***Answer:*** We agree with the reviewer that the language is a bit confusing and have changed the line to say "by up to 2 orders of magnitude" although the improvement is slightly larger than 2 orders of magnitude in the best case. We feel this point is emphasized enough in the Section labeled "Computational results" so we are happy to change the language here to be more clear.

# References

[1] Safety and pharmacokinetics of broadly neutralising human monoclonal antibody vrc07-523ls in healthy adults: a phase 1 dose-escalation clinical trial. *The Lancet HIV*, 6(10):e667 – e679, 2019.

[2] Darlene Barnard, Huaiyu Sun, Leroy Baker, and Mark S. Marshall. In vitro inhibition of ras–raf association by short peptides. *Biochemical and Biophysical Research Communications*, 247(1):176 – 180, 1998.

[3] Jeffrey R. Brender and Yang Zhang. Predicting the Effect of Mutations on Protein-Protein Binding Interactions through Structure-Based Interface Profiles. *PLoS Computational Biology*, 11(10):e1004494, oct 2015.

[4] Greg Buhrman, Casey OConnor, Brandon Zerbe, Bradley M. Kearney, Raeanne Napoleon, Elizaveta A. Kovrigina, Sandor Vajda, Dima Kozakov, Evgenii L. Kovrigin, and Carla Mattos. Analysis of binding site hot spots on the surface of ras gtpase. *Journal of Molecular Biology*, 413(4):773 – 789, 2011.

[5] F-X Campbell-Valois, Kirill Tarassov, and SW Michnick. Massive sequence perturbation of the Raf Ras binding domain reveals relationships between sequence conservation, secondary structure propensity, hydrophobic core organization and stability. *Journal of molecular biology*, 362(1):151–171, 2006.

[6] Mehmet Cetin, William E. Evenson, Garrett G. Gross, Farzad Jalali-Yazdi, Daniel Krieger, Don Arnold, Terry T. Takahashi, and Richard W. Roberts. Rasins: Genetically encoded intrabodies of activated ras proteins. *Journal of Molecular Biology*, 429(4):562 – 573, 2017. Molecular Mechanisms of Autophagy, Part B.

[7] Cheng-Yu Chen, Ivelin Georgiev, Amy C Anderson, and Bruce R Donald. Computational structure-based redesign of enzyme activity. *Proc Natl Acad Sci USA*, 106(10):3764–9, Mar 2009.

[8] Daniel Filchtinski, Oz Sharabi, Alma Rüppel, Ingrid R Vetter, Christian Herrmann, and Julia M Shifman. What makes Ras an efficient molecular switch: a computational, biophysical, and structural study of Ras-GDP interactions with mutants of Raf. *Journal of molecular biology*, 399(3):422–435, 2010.

[9] Kathleen M Frey, Ivelin Georgiev, Bruce R Donald, and Amy C Anderson. Predicting resistance mutations using protein design algorithms. *Proc Natl Acad Sci U S A*, 107(31):13707–12, Aug 2010.

[10] Masha Fridman, Hiroshi Maruta, Jorge Gonez, Francesca Walker, Herbert Treutlein, Jun Zeng, and Antony Burgess. Point mutants of c-raf-1 RBD with elevated binding to v-Ha-Ras. *Journal of Biological Chemistry*, 275(39):30363–30371, 2000.

[11] Masha Fridman, Anjali Tikoo, Mikael Varga, Anne Murphy, MS Nur-E-Kamal, and Hiroshi Maruta. The minimal fragments of c-Raf-1 and NF1 that can suppress v-Ha-Ras-induced malignant phenotype. *Journal of Biological Chemistry*, 269(48):30105–30108, 1994.

[12] Masha Fridman, Francesca Walker, Bruno Catimel, Teresa Domagala, Edouard Nice, and Antony Burgess. c-Raf-1 RBD associates with a subset of active vH-Ras. *Biochemistry*, 39(50):15603–15611, 2000.

[13] I. Georgiev, S. Schmidt, Y. Li, D. Wycuff, G. Ofek, N. Doria-Rose, T. Luongo, Y. Yang, T. Zhou, B. R. Donald, J. Mascola, and P. Kwong. Design of epitope-specific probes for sera analysis and antibody isolation. *Retrovirology*, 9, 2012.

[14] Ivelin Georgiev and Bruce R Donald. Dead-end elimination with backbone flexibility. *Bioinformatics*, 23(13):i185–94, Jul 2007.

[15] Ivelin Georgiev, Ryan H Lilien, and Bruce R Donald. The minimized dead-end elimination criterion and its application to protein redesign in a hybrid scoring and search algorithm for computing partition functions over molecular ensembles. *J Comput Chem*, 29(10):1527–42, Jul 2008.

[16] Ivelin S Georgiev, Rebecca S Rudicell, Kevin O Saunders, Wei Shi, Tatsiana Kirys, Krisha McKee, Sijy O'Dell, Gwo-Yu Chuang, Zhi-Yong Yang, Gilad Ofek, Mark Connors, John R Mascola, Gary J Nabel, and Peter D Kwong. Antibodies VRC01 and 10E8 neutralize HIV-1 with high breadth and potency even with IG-framework regions substantially reverted to germline. *J Immunol*, 192(3):1100–6, Feb 2014.

[17] Michael J Gorczynski, Jolanta Grembecka, Yunpeng Zhou, Yali Kong, Liya Roudaia, Michael G Douvas, Miki Newman, Izabela Bielnicka, Gwen Baber, Takeshi Corpora, Jianxia Shi, Mohini Sridharan, Ryan Lilien, Bruce R Donald, Nancy A Speck, Milton L Brown, and John H Bushweller. Allosteric inhibition of the protein-protein interaction between the leukemia-associated proteins Runx1 and CBFbeta. *Chem Biol*, 14(10):1186–97, Oct 2007.

[18] Christine Gorman, Richard H Skinner, Jane V Skelly, Stephen Neidle, and Peter N Lowe. Equilibrium and kinetic measurements reveal rapidly reversible binding of Ras to Raf. *Journal of Biological Chemistry*, 271(12):6713–6719, 1996.

[19] Sandrine Guillard, Paulina Kolasinska-Zwierz, Judit Debreczeni, Jason Breed, Jing Zhang, Nicolas Bery, Rose Marwood, Jon Tart, Ross Overman, Pawel Stocki, Bina Mistry, Christopher Phillips, Terence Rabbitts, Ronald Jackson, and Ralph Minter. Structural and functional characterization of a darpin which inhibits ras nucleotide exchange. *Nature Communications*, 8(1):16111, 2017.

[20] Mark A Hallen and Bruce R Donald. COMETS (constrained optimization of multistate energies by tree search): A provable and efficient protein design algorithm to optimize binding affinity and specificity with respect to sequence. *Journal of Computational Biology*, 23(5):311–321, 2016.

[21] Mark A Hallen and Bruce R Donald. CATS (coordinates of atoms by taylor series): protein design with backbone flexibility in all locally feasible directions. *Bioinformatics*, 33(14):i5–i12, Jul 2017.

[22] Mark A Hallen, Daniel A Keedy, and Bruce R Donald. Dead-end elimination with perturbations (DEEPer): a provable protein design algorithm with continuous sidechain and backbone flexibility. *Proteins*, 81(1):18–39, Jan 2013.

[23] Mark A. Hallen, Jeffrey W. Martin, Adegoke Ojewole, Jonathan D. Jou, Anna U. Lowegard, Marcel S. Frenkel, Pablo Gainza, Hunter M. Nisonoff, Aditya Mukund, Siyu Wang, Graham T. Holt, David Zhou, Elizabeth Dowd, and Bruce R. Donald. OSPREY 3.0: Open-source protein redesign for you, with powerful new features. *Journal of Computational Chemistry*, 39(30):2494–2507, 2018.

[24] Christian Herrmann, Gudrun Horn, Marcel Spaargaren, and Alfred Wittinghofer. Differential interaction of the ras family GTP-binding proteins H-Ras, Rap1A, and R-Ras with the putative effector molecules Raf kinase and Ral-guanine nucleotide exchange factor. *Journal of Biological Chemistry*, 271(12):6794–6800, 1996.

[25] Graham T. Holt, Jonathan D. Jou, Nicholas P. Gill, Anna U. Lowegard, Jeffrey W. Martin, Dean R. Madden, and Bruce R. Donald. Computational analysis of energy landscapes reveals dynamic features that contribute to binding of inhibitors to cftr-associated ligand. *The Journal of Physical Chemistry B*, 123(49):10441–10455, 2019. PMID: 31697075.

[26] Monique J. Kauke, Michael W. Traxlmayr, Jillian A. Parker, Jonathan D. Kiefer, Ryan Knihtila, John McGee, Greg Verdine, Carla Mattos, and K. Dane Wittrup. An engineered protein antagonist of k-ras/b-raf interaction. *Scientific Reports*, 7(1):5831, 2017.

[27] Christina Kiel, Daniel Filchtinski, Michael Spoerner, Gideon Schreiber, Hans Robert Kalbitzer, and Christian Herrmann. Improved binding of Raf to Ras·GDP is correlated with biological activity. *Journal of Biological Chemistry*, 284(46):31893–31902, 2009.

[28] Christina Kiel, Luis Serrano, and Christian Herrmann. A detailed thermodynamic analysis of ras/effector complex interfaces. *Journal of Molecular Biology*, 340(5):1039 – 1058, 2004.

[29] Latesh Lad, Sheila Clancy, Maria Kovalenko, Chian Liu, Terence Hui, Victoria Smith, and Nikos Pagratis. High-throughput kinetic screening of hybridomas to identify high-affinity antibodies using bio-layer interferometry. *Journal of biomolecular screening*, 20(4):498–507, 2015.

[30] Ryan H Lilien, Brian W Stevens, Amy C Anderson, and Bruce R Donald. A novel ensemble-based scoring and search algorithm for protein redesign and its application to modify the substrate specificity of the gramicidin synthetase a phenylalanine adenylation enzyme. *J Comput Biol*, 12(6):740–61, 2005.

[31] Frank McCormick. Targeting KRAS directly. *Annual Review of Cancer Biology*, 2:81–90, 2018.

[32] Helen R. Mott and Darerca Owen. Structures of ras superfamily effector complexes: What have we learnt in two decades? *Critical Reviews in Biochemistry and Molecular Biology*, 50(2):85–133, Mar 2015.

[33] Nicolas Nassar, Gudrun Horn, Christian A. Herrmann, Anna Scherer, Frank McCormick, and Alfred Wittinghofer. The 2.2 å crystal structure of the ras-binding domain of the serine/threonine kinase c-raf1 in complex with rapla and a gtp analogue. *Nature*, 375(6532):554–560, 1995.

[34] Adegoke Ojewole, Anna Lowegard, Pablo Gainza, Stephanie M Reeve, Ivelin Georgiev, Amy C Anderson, and Bruce R Donald. OSPREY predicts resistance mutations using positive and negative computational protein design. In *Computational Protein Design*, chapter 15, pages 291–306. Springer, 2017.

[35] E. F. Pai, U. Krengel, G. A. Petsko, R. S. Goody, W. Kabsch, and A. Wittinghofer. Refined crystal structure of the triphosphate conformation of h-ras p21 at 1.35 a resolution: implications for the mechanism of gtp hydrolysis. *The EMBO journal*, 9(8):2351–2359, Aug 1990. 2196171[pmid].

[36] Samantha Perspicace, David Banner, Jörg Benz, Francis Müller, Daniel Schlatter, and Walter Huber. Fragment-based screening using surface plasmon resonance technology. *Journal of biomolecular screening*, 14(4):337–349, 2009.

[37] Stephanie M Reeve, Pablo Gainza, Kathleen M Frey, Ivelin Georgiev, Bruce R Donald, and Amy C Anderson. Protein design algorithms predict viable resistance to an experimental antifolate. *Proc Natl Acad Sci U S A*, 112(3):749–54, Jan 2015.

[38] Kyle E Roberts, Patrick R Cushing, Prisca Boisguerin, Dean R Madden, and Bruce R Donald. Computational design of a PDZ domain peptide inhibitor that rescues CFTR activity. *PLoS Comput Biol*, 8(4):e1002477, 2012.

[39] Rebecca S Rudicell, Young Do Kwon, Sung-Youl Ko, Amarendra Pegu, Mark K Louder, Ivelin S Georgiev, Xueling Wu, Jiang Zhu, Jeffrey C Boyington, Xuejun Chen, Wei Shi, Zhi-Yong Yang, Nicole A Doria-Rose, Krisha McKee, Sijy O'Dell, Stephen D Schmidt, Gwo-Yu Chuang, Aliaksandr Druz, Cinque Soto, Yongping Yang, Baoshan Zhang, Tongqing Zhou, John-Paul Todd, Krissey E Lloyd, Joshua Eudailey, Kyle E Roberts, Bruce R Donald, Robert T Bailer, Julie Ledgerwood, NISC Comparative Sequencing Program, James C Mullikin, Lawrence Shapiro, Richard A Koup, Barney S Graham, Martha C Nason, Mark Connors, Barton F Haynes, Srinivas S Rao, Mario Roederer, Peter D Kwong, John R Mascola, and Gary J Nabel. Enhanced potency of a broadly neutralizing HIV-1 antibody in vitro improves protection against lentiviral infection in vivo. *J Virol*, 88(21):12669–82, Nov 2014.

[40] Gideon Schreiber and Amy E Keating. Protein binding specificity versus promiscuity. *Current Opinion in Structural Biology*, 21(1):50 – 61, 2011.

[41] David Simoncini, David Allouche, Simon de Givry, Céline Delmas, Sophie Barbe, and Thomas Schiex. Guaranteed discrete energy optimization on large protein design problems. *J Chem Theory Comput*, 11(12):5980–9, Dec 2015.

[42] Russell Spencer-Smith and John P. O'Bryan. Direct inhibition of ras: Quest for the holy grail? *Seminars in Cancer Biology*, 54:138–148, 2019.

[43] Brian W Stevens, Ryan H Lilien, Ivelin Georgiev, Bruce R Donald, and Amy C Anderson. Redesigning the PheA domain of gramicidin synthetase leads to a new understanding of the enzyme's mechanism and selectivity. *Biochemistry*, 45(51):15495–504, Dec 2006.

[44] Renee Tobias and Sriram Kumaraswamy. Biomolecular binding kinetics assays on the octet platform. Technical report, ForteBio. https://www.fortebio.com/sites/default/files/en/assets/app-note/biomolecular-binding-kinetics-assays-on-the-octet-platform.pdf.

[45] Thi B. Trinh, Punit Upadhyaya, Ziqing Qian, and Dehua Pei. Discovery of a direct ras inhibitor by screening a combinatorial library of cell-permeable bicyclic peptides. *ACS Combinatorial Science*, 18(1):75–85, Jan 2016.

[46] Punit Upadhyaya, Ziqing Qian, Nicholas G. Selner, Sarah R. Clippinger, Zhengrong Wu, Roger Briesewitz, and Dehua Pei. Inhibition of ras signaling by blocking ras-effector interactions with cyclic peptides. *Angewandte Chemie International Edition*, 54(26):7602–7606, Jun 2015.

[47] Xianghong Wu, Punit Upadhyaya, Miguel A. Villalona-Calero, Roger Briesewitz, and Dehua Pei. Inhibition of ras-effector interactions by cyclic peptides. *MedChemComm*, 4(2):378–382, 2013.

[48] Peng Xiong, Chengxin Zhang, Wei Zheng, and Yang Zhang. Bindprofx: Assessing mutation-induced binding affinity change by protein interface profiles with pseudo-counts. *Journal of Molecular Biology*, 429(3):426 – 434, 2017. Computation Resources for Molecular Biology.

[49] Francisco Ylera, Stefan Harth, Dirk Waldherr, Christian Frisch, and Achim Knappik. Off-rate screening for selection of high-affinity anti-drug antibodies. *Analytical biochemistry*, 441(2):208–213, 2013.
