# Supplementary material for: Novel, provable algorithms for efficient ensemble-based computational protein design and their application to the redesign of the c-Raf-RBD:KRas protein-protein interface
Source: PLoS Comput Biol. 2020 Jun 8;16(6):e1007447. doi: 10.1371/journal.pcbi.1007447 (PMC7329130; doi:10.1371/journal.pcbi.1007447)
Supplement: Attachment — Submitted filename: RTR_3.pdf [file pcbi.1007447.s014.pdf]

# How we responded to reviewers' comments: Novel, Provable Algorithms for Efficient Ensemble-Based Computational Protein Design and Their Application to the Redesign of the c-Raf-RBD:KRas Protein-Protein Interface

Anna U. Lowegard Marcel S. Frenkel Graham T. Holt
Jonathan D. Jou Adegoke A. Ojewole Bruce R. Donald

We found the editors' and reviewers' comments very helpful, and we thank them for their suggestions which have greatly improved our manuscript. Below, we address each comment individually and summarize the resulting changes to the manuscript. Major changes to the revised manuscript are indicated in red. References (e.g., [1]) can be found at the end of this response.

## Editors

> Both reviewers recommended publication. The second reviewer had some concerns about whether all the necessary data are presented in the paper.

***Answer:*** We thank the editors for their response, and have included all necessary data.

> S/he suggests some work that might be outside the scope of the paper, and we think that does not need to be included in your final revision. But please respond to the other suggestions in a minor revision.

***Answer:*** We agree that some suggested experiments may be outside the scope of this manuscript, and would be better suited for a separate study. We hope such work could be performed for a future paper.

# Reviewer 1

Some of the criticisms I raised before are still relevant to this revision: prospective validation is done on just one single-point mutant and the results are modest compared to data presented in recent years for protein design methods. Nevertheless, the authors made a very serious effort to clarify the message and provide more detail on the calculations and the experiments while also toning down some of the language that seemed be carried away in the original submission.

I think that the major contribution of this paper is in the very extensive theoretical treatment. Further experimental validation including, possibly, a side-by-side comparison of this method with others may provide the answer to the questions I raised on the method's scope.

In summary, I would like to congratulate the authors on this work and also on their sincere efforts to address my previous comments which may have been phrased too harshly (my apologies for that).

***Answer:*** We thank the reviewer for their comments, and are pleased that their original comments and our responses have improved the manuscript. We agree that a comparison between this method and others would be valuable future work. We hope such work could be performed for a future paper.

# Reviewer 2

I very much like this paper and would like to see it published soon. I think there are a handful of relatively small things that should be addressed that do not necessarily require another round of review.

I think the objections made by the first reviewer to the original submission were well deserved and that the changes that were made to the text in response have improved the manuscript considerably. Removing the repetitive emphasis on provability made the paper much more enjoyable to read.

***Answer:*** We thank the reviewer for their kind comments, and are glad that our responses have improved the manuscript.

I also think the point about using Spearman's rho instead of Pearson's r is still important and was not well addressed in the revision: the authors are not reporting r presumably because the T68K outlier makes r look quite bad. I can only guess that the authors suspect a reader would distill the paper to a single r value and look past Osprey as a tool for their project. I wanted to compute an r value myself looking to the data in Supp Table 3, but while the upper and lower bounds on log(K) for each mutation + wt is given, that does not readily translate into the delta-b value that is

presented in Figure 5. Another column for delta-b would be nice. Also missing from Supp Table 3 is the WT dG values (where available) that a reader would need to compute r accurately.

***Answer:*** We agree that the necessary data was not initially provided and was an oversight on our part. We have updated Table 2 in the supporting information to include this necessary data which includes experimentally reported $K_a$ values and experimentally reported percent change in binding. We have also calculated a Pearson's $r$ (0.64) directly comparing the change in binding upon mutation from wild-type between the experimentally reported values and those predicted by OSPREY. In the revised S2 Table, we report change in relative binding for the previously reported mutants. For the majority of the previously reported mutants, this is all that is provided in those publications, so this is the available data. For the subset of publications that do report a $K_d$, we report that in the table, and we calculate a change in relative binding from those values, and we include that in the table as well.

A paper where the authors look into how well K* with Osprey's energy function performs compared to other techniques for binding energy calculations (e.g. FoldX) is definitely needed, but perhaps is beyond the scope of this work.

***Answer:*** We agree that such a study would be an interesting and worthwhile endeavor, and thank the reviewer for the suggestion. We hope such work could be performed for a future paper.

On this topic, I would also add that I find the sentence explaining why K* does not do a good job predicting absolute values of binding energies

> "since our current designs likely underestimate entropic contributions to binding upon mutation due to various limitations in biological modeling"

to be very unsatisfying! I was under the impression that K* would do a much better job than other techniques for estimating mutation ddGs because it considers side-chain entropic contributions explicitly. If entropy is poorly estimated by EWAK*, wouldn't it be estimated even more poorly by other molecular modeling applications? Finally, what aspect of entropy do you suspect is under-considered? ("various" doesn't help me). One question that perhaps could be addressed in a subsequent ddG comparison paper is: how well does K* (including FRIES and EWAK) perform using a different energy function? (Can the energy function that Osprey is using be swapped out with another energy function such as the one used in FoldX?)

***Answer:*** The reviewer raises an excellent point. $K^*$ does account for more entropy than other, GMEC-based methods [1, 3, 7, 9, 11, 14, 17] by considering side-chain conformational entropy. We do expect that $K^*$ would do a better job predicting ddGs than methods that do

not consider side-chain configurational entropy, and have in fact found side-chain entropy to be necessary to find top empirical design results [2,8,15,16]. The value of accurately modeling side chain configurational entropy for protein-protein interfaces is also supported by previous work by Sarel Fleishman and David Baker [4]. However, side-chain configurational entropy is only a subset of biophysically relevant entropy. Our statement was intended to compare the entropy considered in our method to the entropy of biological systems, not to entropy considered in other computational methods.

We suspect that solvent entropy, backbone entropy, and entropy from methyl rotations are under-considered by our method. While our energy function does contain an implicit solvent term, we suspect that it is less accurate than explicit solvent calculations [18]. The OSPREY suite does contain methods to consider backbone flexibility [5, 6, 10, 13], but these primarily model small, continuous backbone movements. Finally, this study did not model continuous methyl rotations, although this will be possible (and likely recommended) in future releases.

In principle any pairwise-decomposable energy function can be used with OSPREY. In addition to the default Amber98 forcefield, previous designs [16] have used the CHARMM19 forcefield. We agree that it would be valuable in the future to systematically test the effects of different energy functions. A step in this direction is given in our paper on EPIC [11], where two alternative forcefields are tested. Non-pairwise energy functions were tested for computational design in [11, 12].

To improve our explanation we have modified the quoted passage to read:

> ... contributions to binding due to solvent entropy, backbone entropy, and rotating methyl groups. Nevertheless, by explicitly modeling side-chain configurational entropy, our method considers more conformational entropy than GMEC-based methods – ...

> I appreciate the clarity that was added to the algorithm description, especially with regard to fact that the partition functions of the three species are approximated separately, and with the energy bound based on the WT sequence that FRIES uses to prune sequences.

***Answer:*** We are glad that our response to the reviewer's comment has improved the manuscript.

> The revision was missing almost all of the figures – only figure 8 of the non-supplemental-figures is included. I had to go back to the original manuscript to find the others.

***Answer:*** We apologize for this difficulty, and would be happy to provide the reviewer with any and all figures, or a version of the updated manuscript that does include all figures. While we did submit all figures with the revision and verified that all figures were visible in

the document that the authors were asked to approve, we suspect that only changed figures were included for you by the online editorial manager system. If this happens again, please ask the editors to contact us. Through the editors we can supply any figures that are not visible to you.

> **Have all data underlying the figures and results presented in the manuscript been provided?** No: The data needed to reconstruct figure 5 is not fully present in supplemental table 3. It is possible for the reader to hunt down all of the previously reported experimentally measured ddGs from the previous literature, but it would be nice for the authors to simply include these numbers. It is less clear how one goes from the upper and lower bounds on the log(K) to the delta-b values that the authors use.

***Answer:*** We agree that the appropriate data was not adequately presented in the supplemental. However, we request that the reviewer please look at supplemental Table 2 not supplemental Table 3. Table 3 corresponds to the prospective designs and Table 2 corresponds to the retrospective designs that are presented in Figure 5. In Table 2 we present the percent change in binding, while $\log_{10}$(% change in binding)-2 is what is plotted in Figure 5. To make this more clear we have added to the language in the supplemental Table 2 along with the values. We have also added any reported $K_a$ values from existing literature as well as the reported percent change in binding and a Pearson's $r$.

In sum, we agree that we did not previously enclose all data presented in figures, but we have enclosed all of the data this time.

# References


[1] Bernard Chazelle, Carl Kingsford, and Mona Singh. A semidefinite programming approach to side chain positioning with new rounding strategies. *INFORMS Journal on Computing*, 16(4):380–392, September 2004.

[2] Cheng-Yu Chen, Ivelin Georgiev, Amy C Anderson, and Bruce R Donald. Computational structure-based redesign of enzyme activity. *Proc Natl Acad Sci USA*, 106(10):3764–9, Mar 2009.

[3] B I Dahiyat and S L Mayo. De novo protein design: fully automated sequence selection. *Science*, 278(5335):82–7, Oct 1997.

[4] Sarel J Fleishman, Sagar D Khare, Nobuyasu Koga, and David Baker. Restricted sidechain plasticity in the structures of native proteins and complexes. *Protein Sci*, 20(4):753–7, Apr 2011.

[5] Ivelin Georgiev and Bruce R Donald. Dead-end elimination with backbone flexibility. *Bioinformatics*, 23(13):i185–94, Jul 2007.

[6] Ivelin Georgiev, Daniel Keedy, Jane S Richardson, David C Richardson, and Bruce R Donald. Algorithm for backrub motions in protein design. *Bioinformatics*, 24(13):i196–204, Jul 2008.

[7] Ivelin Georgiev, Ryan H Lilien, and Bruce R Donald. Improved pruning algorithms and divide-and-conquer strategies for dead-end elimination, with application to protein design. *Bioinformatics*, 22(14):e174–83, Jul 2006.

[8] Ivelin Georgiev, Ryan H Lilien, and Bruce R Donald. The minimized dead-end elimination criterion and its application to protein redesign in a hybrid scoring and search algorithm for computing partition functions over molecular ensembles. *J Comput Chem*, 29(10):1527–42, Jul 2008.

[9] Mark A Hallen and Bruce R Donald. CATS (coordinates of atoms by taylor series): protein design with backbone flexibility in all locally feasible directions. *Bioinformatics*, 33(14):i5–i12, Jul 2017.

[10] Mark A. Hallen and Bruce R. Donald. CATS (Coordinates of Atoms by Taylor Series): Protein design with backbone flexibility in all locally feasible directions. *Bioinformatics*, 33(14):i5–i12, 2017.

[11] Mark A Hallen, Pablo Gainza, and Bruce R Donald. Compact representation of continuous energy surfaces for more efficient protein design. *J Chem Theory Comput*, 11(5):2292–306, May 2015.

[12] Mark A Hallen, Jonathan D Jou, and Bruce R Donald. LUTE (Local Unpruned Tuple Expansion): Accurate Continuously Flexible Protein Design with General Energy Functions and Rigid Rotamer-Like Efficiency. *Journal of Computational Biology*, 24(6):536–546, 2017.

[13] Mark A Hallen, Daniel A Keedy, and Bruce R Donald. Dead-end elimination with perturbations (DEEPer): a provable protein design algorithm with continuous sidechain and backbone flexibility. *Proteins*, 81(1):18–39, Jan 2013.

[14] A R Leach and A P Lemon. Exploring the conformational space of protein side chains using dead-end elimination and the a* algorithm. *Proteins*, 33(2):227–39, Nov 1998.

[15] Ryan H Lilien, Brian W Stevens, Amy C Anderson, and Bruce R Donald. A novel ensemble-based scoring and search algorithm for protein redesign and its application to modify the substrate specificity of the gramicidin synthetase a phenylalanine adenylation enzyme. *J Comput Biol*, 12(6):740–61, 2005.

[16] Kyle E Roberts, Patrick R Cushing, Prisca Boisguerin, Dean R Madden, and Bruce R Donald. Computational design of a PDZ domain peptide inhibitor that rescues CFTR activity. *PLoS Comput Biol*, 8(4):e1002477, 2012.

[17] Seydou Traoré, David Allouche, Isabelle André, Simon de Givry, George Katsirelos, Thomas Schiex, and Sophie Barbe. A new framework for computational protein design through cost function network optimization. *Bioinformatics*, 29(17):2129–36, Sep 2013.

[18] Jin Zhang, Haiyang Zhang, Tao Wu, Qi Wang, and David van der Spoel. Comparison of implicit and explicit solvent models for the calculation of solvation free energy in organic solvents. *Journal of Chemical Theory and Computation*, 13(3):1034–1043, 2017. PMID: 28245118.
